# Memory consolidation reconfigures neural pathways involved in the suppression of emotional memories

**DOI:** 10.1038/ncomms13375

**Published:** 2016-11-29

**Authors:** Yunzhe Liu, Wanjun Lin, Chao Liu, Yuejia Luo, Jianhui Wu, Peter J. Bayley, Shaozheng Qin

**Affiliations:** 1 State Key Laboratory of Cognitive Neuroscience and Learning & IDG/McGovern Institute for Brain Research, Beijing Normal University, Beijing 100875, China; 2 Institute of Affective and Social Neuroscience & College of Psychology and Sociology, Shenzhen University, Shenzhen 518060, China; 3 Shenzhen Institute of Neuroscience, Shenzhen 518057, China; 4 War Related Illness and Injury Study Center, US Department of Veterans Affairs, Palo Alto, California 94304, USA; 5 Department of Psychiatry and Behavioral Sciences, Stanford University School of Medicine, Stanford, California 94304, USA

## Abstract

The ability to suppress unwanted emotional memories is crucial for human mental health. Through consolidation over time, emotional memories often become resistant to change. However, how consolidation impacts the effectiveness of emotional memory suppression is still unknown. Using event-related fMRI while concurrently recording skin conductance, we investigated the neurobiological processes underlying the suppression of aversive memories before and after overnight consolidation. Here we report that consolidated aversive memories retain their emotional reactivity and become more resistant to suppression. Suppression of consolidated memories involves higher prefrontal engagement, and less concomitant hippocampal and amygdala disengagement. In parallel, we show a shift away from hippocampal-dependent representational patterns to distributed neocortical representational patterns in the suppression of aversive memories after consolidation. These findings demonstrate rapid changes in emotional memory organization with overnight consolidation, and suggest possible neurobiological bases underlying the resistance to suppression of emotional memories in affective disorders.

## Abstract (web summary)

As memories consolidate over time, they become resistant to change, though how this impacts the volitional suppression of memories is not known. Liu and colleagues show that, after overnight consolidation, aversive memories exhibit distributed prefrontal representations and are harder to suppress.

## Graphical abstract

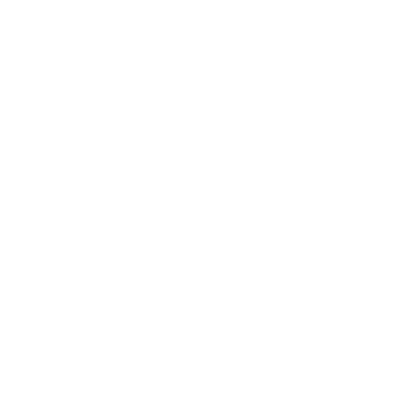

Our memories for aversive or traumatic events are often vivid and long lasting relative to that of non-aversive or neutral experiences. The enhancement of aversive memory is thought to be due to autonomic reactions to the emotional charge stimulating the encoding and subsequent consolidation of what is referred to as an ‘emotional memory'^1^,^2^. Although emotional memories are enduring, they can, to some extent, be consciously controlled through voluntary suppression in healthy individuals^3^,^4^. A failure to suppress unwanted memories has been linked to symptoms in a number of psychiatric disorders including the ruminative state found in depression and intrusive memories in posttraumatic stress disorder (PTSD)^5^,^6^,^7^,^8^. Over time, emotional memories often become more resistant to suppression most likely through a process of consolidation in which sleep is thought to play a vital role^7^,^9^,^10^. However, it remains unknown how consolidation impacts the effectiveness of voluntary suppression of unwanted emotional memories.

Laboratory studies of the neural basis of memory suppression often use a suppression-induced or motivated forgetting paradigm—the ‘Think/NoThink (TNT)' procedure^3^,^4^,^11^, in which inhibitory control of a newly acquired memory is assessed by the compromised ability to recall it at a later time point^3^,^12^. Experiments have shown memory suppression to involve right dorsolateral prefrontal cortex (DLPFC) activation and concomitant reduced hippocampal engagement^11^,^13^, as well as reduced amygdala activity for emotional memories^4^,^5^. In other words, prefrontal inhibitory control over the hippocampal memory and amygdala emotional systems is believed to play a crucial role in voluntary suppression of emotional memories. However, previous studies of memory suppression typically involve materials that are acquired and recalled within minutes or a few hours. In reality, most emotional memories involve events that occurred days, months or years ago. Such memories are expected to be stabilized and assimilated into long-term memory system through consolidation processes^1^,^14^,^15^.

Many models of memory consolidation suggest that newly acquired memories are initially dependent on the hippocampus and surrounding medial temporal lobe (MTL) structures, and gradually become dependent on a network of cortical regions^1^,^15^,^16^,^17^. Critically, newly acquired memories are labile and susceptible to change^11^,^18^,^19^. Over time they become stabilized in the neocortex through consolidation^14^,^15^. Memory consolidation involves reorganization at both the synaptic and systems levels^20^. Synaptic consolidation is thought to be complete within hours following learning, and involves the stabilization of synaptic connectivity in local circuits^21^. By contrast, systems consolidation is a more prolonged process and involves gradual reorganization of the brain regions that support long-term memory^1^,^15^. The timecourse of memory consolidation at the systems level appears to be in the order of several years^15^. Notably, recent human neuroimaging studies suggest that consolidation can lead to measurable changes over a 24-h period^22^. Such findings are consistent with the notion that systems consolidation depends at least in part on overnight sleep^7^,^9^. Thus, memories following overnight sleep may undergo notable changes in functional organization between the hippocampus and distributed neocortical regions^22^. As a result, distinct mechanims may be involved in the suppression of consolidated memories following overnight sleep. However, we know very little about how overnight consolidation impacts brain functional pathways underlying voluntary suppression of emotional memories.

We addressed this question using an event-related functional magnetic resonance imaging (fMRI) memory suppression task coupled with overnight consolidation, which included memory acquisition, TNT and post-scan testing phases (Fig. 1a–c). During the acquisition phase, participants were trained to remember two sets of associations between faces and aversive scenes on two consecutive days, which occurred about 24 h and 30 min before fMRI scanning. During the Think/NoThink phase, participants underwent fMRI while performing the ‘TNT' task^2^,^3^, in which faces served as cues, with half of the cues learnt 30-min before (that is, newly acquired condition) and the other half learnt 24 h (that is, overnight consolidation condition) before scanning. Skin conductance responses (SCRs) were recorded simultaneously with fMRI scanning to provide an on-line monitoring of physiological reactivity associated with aversive memories. During the post-scan testing phase, participants performed a cued-recall memory test to validate suppression-induced forgetting. Notably, behavioural and physiological data from another independent cohort of 25 participants were used for replication purposes to confirm the stability and robustness of the observed effects of overnight consolidation on suppression of aversive memories. An additional behavioural control experiment with face and neutral stimuli was conducted with another independent 30 participants to investigate the effects of overnight consolidation on suppression of neutral memories.

To further examine the neural representations associated with individual memories before and after overnight consolidation, we implemented a novel analytic approach based on multivariate pattern dissimilarity of item-specific neural activity^23^,^24^,^25^. This method has been used to identify the representational patterns of neural population codes by means of representational distance matrices^24^,^26^, and provides a unique way to link neuronal activity with representational content of the brain's memory information processing^19^,^27^,^28^,^29^. We show that aversive memories after overnight consolidation become more resistant to suppression, as evidenced by less suppression-induced forgetting and enduring SCR levels. Suppression of consolidated aversive memories, relative to newly acquired ones, appears to involve higher prefrontal inhibitory engagement, but accompanied with less concomitant hippocampal and amygdala disengagement. These effects are paralleled with a shift away from hippocampal-dependent representational patterns to distributed neocortical representational patterns in suppression of aversive memories after overnight consolidation. Altogether, our findings provide converging evidence to support that overnight consolidation assimilates emotional memories into more distributed representational patterns in the neocortex, thus making these memories more resistant to suppression through prefrontal-hippocampal inhibitory pathway.

## Results

### Enduring physiological responses after consolidation

**Figure 1:**
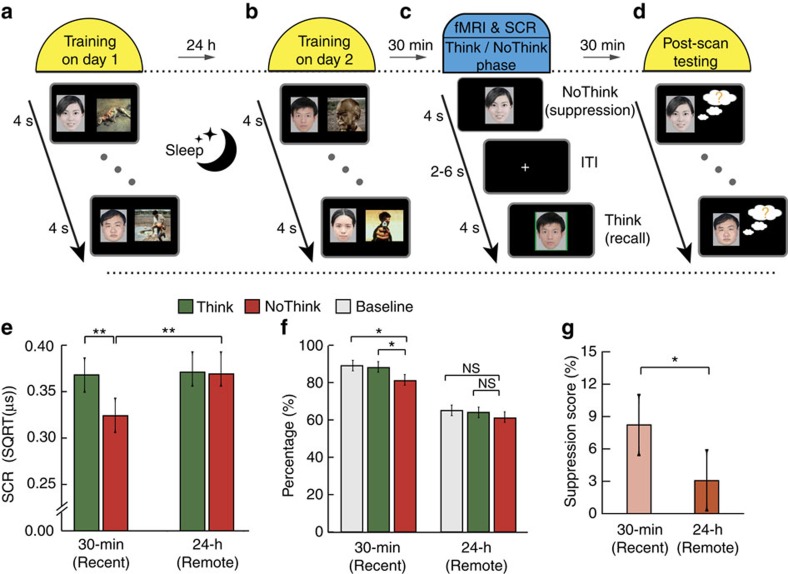
Experimental design and results from physiology and behaviour. (**a**,**b**) The experiment consisted of three phases, including acquisition, Think/NoThink and post-scan memory test. During the acquisition phase outside the scanner, participants performed two training sessions on day 1 and day 2, which occurred 24 h and 30 min before the Think/NoThink phase, respectively. Participants were trained to memorize 26 pairs in each of the two acquisition sessions. (**c**) During the Think/NoThink phase, participants underwent fMRI with concurrent recording of skin conductance responses (SCR) while they were performing a ‘Think/NoThink' task. (**d**) During the test phase, participants were given faces as cues and asked to recall their associated target pictures. Behavioural suppression scores were calculated separately for newly acquired (that is, 30-min) and overnight (that is, 24-h) aversive memories based on the difference in memory accuracy between their respective baseline items and NoThink items accordingly. A total number of 18 participants were included in the final analysis. (**e**) Bar graphs depict SCR associated with ‘Think' (green) and ‘NoThink' (red) conditions for aversive memories acquired either 24 h or 30 min ago (referred to as remote or recent memories when appropriate). (**f**) Bar graphs depict cued-recall accuracy for newly acquired and consolidated aversive memories as a function of ‘Think', ‘NoThink' and ‘Baseline' (gray) during the test phase. (**g**) Bar graphs depict suppression scores for both newly acquired and overnight consolidated aversive memories. Error bars represent standard error of mean. *P<0.05; **P<0.01.

First, we examined physiological responses associated with suppression of aversive memories before and after overnight consolidation. A 2-by-2 repeated-measures analysis of variance (ANOVA) on SCR data, with Suppression (NoThink versus Think) and Time (30 min versus 24 h, referred to as Recent versus Remote), revealed main effects of Suppression (*F*(1, 17)=5.79, *P*=0.028, 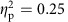) and Time (*F*(1, 17)=6.42, *P*=0.021, 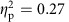). Importantly, we observed a significant Suppression-by-Time interaction (*F*(1, 17)=7.08, *P*=0.016, 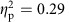). *Post hoc* comparisons using two-tailed paired *t*-tests revealed that suppression of newly acquired aversive memories led to significantly reduced SCR levels relative to Think condition (*t*(17)=−3.10, *P*=0.007, *d*_av_=0.50). This reduction, however, was not found in the suppression of aversive memories following overnight consolidation (*t*(17)=0.14, *P*=0.89, power=0.05; Fig. 1e). To mitigate potential confounds due to a difference in general performance between newly acquired and overnight memories, we performed a subsequent SCR analysis by taking random sub-samplings from 30-min conditions to artificially match the number of trials in 24-h conditions. We replicated our results and found that suppression was indeed accompanied by reduced SCR in newly acquired memories (*t*(17)=−3.33, *P*=0.004, *d*_av_=0.74), but not for overnight aversive memories (*t*(17)=0.02, *P*=0.97, power=0.05; Supplementary Fig. 1). Remarkably, a similar pattern of results was observed in another independent cohort of 25 participants (Supplementary Fig. 2a). To further investigate whether our observed effects were specific to suppression of aversive memories, we conducted an additional control experiment with face and neutral pictures as stimuli in another 30 participants (data from one participant was excluded due to no SCR recording, resulting in 29 participants in total) (Supplementary Fig. 3a–c). Repeated measures ANOVAs revealed neither the main effect of Suppression nor Time-by-Suppression interaction (all *F*(1, 28)<1.50, *P*>0.30, 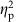
<0.05; Supplementary Fig. 3d), and a generally lower SCR level associated with neutral than aversive memories (mean±s.d, 0.15±0.07 versus 0.36±0.07). Altogether, these results indicate that the reduction in physiological responses to emotional memory reactivity is attenuated over a 24-h time period.

### Less efficient memory suppression after consolidation

Next, we examined the effectiveness of memory suppression after overnight consolidation compared with newly acquired memories. Note that the type of effectiveness under consideration is referred to the mnemonic aftereffects of suppression. A 2-by-2 ANOVA on subsequent memory performance revealed main effects of Suppression (*F*(1, 17)=4.60, *P*=0.047, 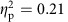) and Time (*F*(1, 17)=14.75, *P*<0.001, 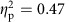; Fig. 1f). Interestingly, we also observed a significant Suppression-by-Time interaction (*F*(1, 17)=5.86, *P*=0.027, 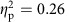) (Fig. 1g). Again, similar results were reproduced in another independent cohort of 25 participants (Supplementary Fig. 2b,c). To examine whether the observed effect was specific to emotional memories, we analysed data from our additional behavioural neutral control experiment. Similar to aversive memories, we found significant main effect of Time (*F*(1, 28)=62.15, *P*<0.001, 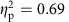) and an interaction between Time and Suppression (*F*(1, 28)=4.89, *P*=0.035, 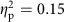; Supplementary Fig. 3e,f). In addition, evidence from our behavioural control experiment with neutral stimuli showed similar effects on the suppression of neutral memories after overnight consolidation (Supplementary Fig. 3e–j). Altogether, these results indicate that overnight consolidation leads to less pronounced suppression-induced forgetting for both emotional and neutral memories.

### Hippocampal–neocortical reorganization after consolidation

**Figure 2:**
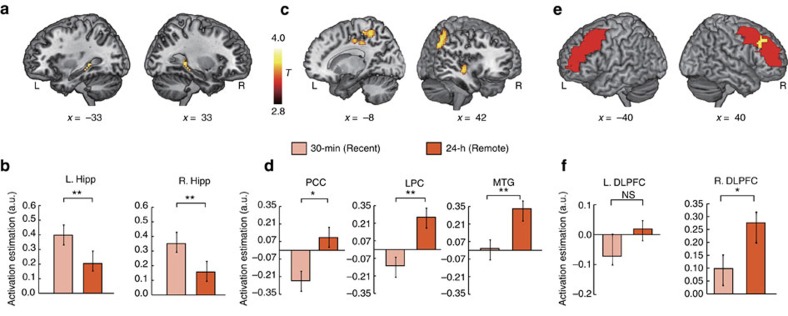
Hippocampal–neocortical functional reorganization after consolidation. (**a**) Left (MNI, peak at −33, −33 and −6) and right hippocampus (MNI, peak at 33, −39 and −6) are involved in retrieval of newly acquired aversive memories in contrast to the overnight consolidation condition (T_30 min>T_24 h), including the left and right posterior hippocampus. (**b**) Bar graphs represent functional activation in the left and right hippocampus showing higher engagement during retrieval of newly acquired relative to consolidated memories. (**c**) Brain regions involved in retrieval of consolidated relative to newly acquired aversive memories (T_30 min<T_24 h), including the right lateral parietal cortex (LPC) and angular gyrus extending into the posterior cingulate cortex (PCC), and the right middle temporal gyrus (MTG). (**d**) Bar graphs represent functional activation in these cortical regions, with higher engagement during retrieval of consolidated relative to newly acquired memories. (**e**,**f**) Bar graphs represent changes in functional engagement of the right and left anatomically defined middle frontal gyrus (that is, DLPFC) during NoThink trials between newly acquired and consolidated memories (NT_30 min<NT_24 h). The significant functional cluster (in ‘hot') in the right DLPFC showing the same effect between Time and Suppression was overlaid on the anatomically defined DLPFC (in ‘red'). Each bar graph was plotted against the fixation period in the TNT task. Colour bars represent *T* values. DLPFC, dorsolateral prefrontal cortex; Hipp, hippocampus; L, left; MFG, middle frontal gyrus; R, right. Error bars represent s.e.m. *P<0.05; **P<0.01; MNI, Montreal Neurological Institute coordinate system.

We then examined functional reorganization of the brain systems involved in retrieval and suppression of aversive memories before and after overnight consolidation. We restricted our analysis to trials that were later remembered and artificially matched the number of trials in the 30 min and 24 h conditions to mitigate potential confounds related to general differences in memory performance and time decay between these conditions. This analysis revealed higher activation in the bilateral hippocampus in the ‘Think' condition of newly acquired memories relative to the overnight consolidation ones (Fig. 2a,b). The ‘Think' condition of aversive memories after overnight consolidation, however, was associated with higher engagement in neocortical regions including lateral parietal cortex (LPC) and angular gyrus extending into posterior cingulate cortex (PCC), and middle temporal gyrus (MTG) (Fig. 2c,d; Supplementary Table 1).

We conducted parallel analyses for all trials irrespective of final recall status to examine general changes in brain systems involved in suppression of aversive memories following overnight consolidation compared with newly acquired condition. We identified a set of widely distributed brain regions previously reported^4^,^11^, including the inferior frontal gyrus, DLPFC and posterior parietal cortex (Supplementary Figs 4,5, Supplementary Table 2). Further analysis of anatomically defined regions of interest (ROIs) revealed significantly higher engagement in the right (*t*(17)=2.32, *P*=0.033, *d*_av_=0.37) but not the left (*t*(17)=1.13, *P*=0.27) DLPFC in suppression of aversive memories after overnight consolidation, compared with newly acquired condition (Fig. 2e,f).

**Figure 3:**
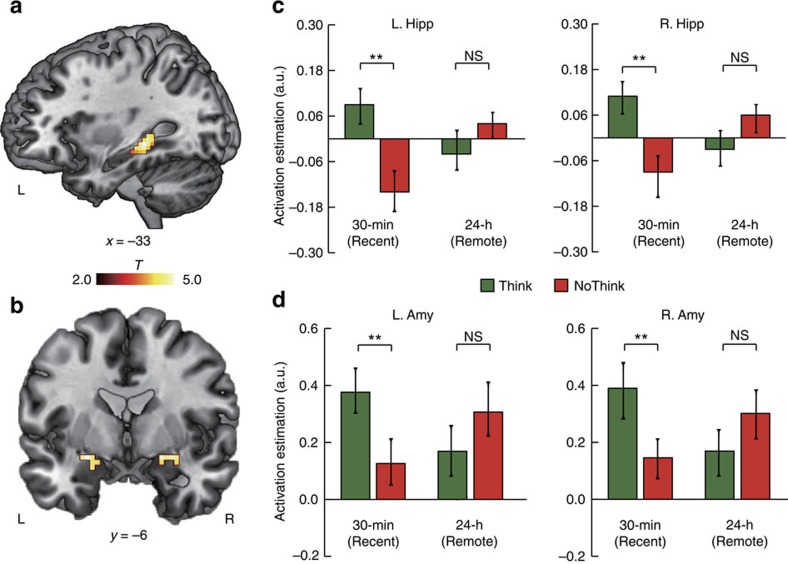
Brain systems underlying memory suppression. (**a**) Sagittal view of significant clusters in the left hippocampus (MNI, peak at −33, −33 and −6), and (**b**) Coronal view of significant clusters in the left (MNI, peak at −24, 0 and −12) and right amygdala (MNI, peak at 21, 0 and −12) showing interaction effects between Suppression and Time (T_30 min—NT_30 min>T_24 h—NT_24 h). (**c**,**d**) Bar graphs represent functional activation in the hippocampus and amygdala. Each bar graph was plotted against the fixation period in the Think/NoThink task. Colour bar represents *T* values and error bars represent standard error of mean (s.e.m.). *P<0.05; **P<0.01; Amy, amygdala; Hipp, hippocampus; L, left; MNI, Montreal Neurological Institute coordinate system; R, right.

To better understand neural mechanisms underlying the effects of overnight consolidation on emotional memory suppression, we contrasted suppression (that is, NoThink trials) with retrieval (that is, Think trials) of aversive memories in overnight consolidation versus the newly acquired condition to explore a Suppression-by-Time interaction effect. This analysis revealed significant clusters in the bilateral hippocampus and amygdala (Fig. 3a,b), and other brain regions (Supplementary Table 3). Follow-up ROI analyses revealed that suppression relative to retrieval of newly acquired aversive memories led to significant reductions in activation of the bilateral hippocampus and amygdala (Fig. 3c,d; all *t*(17)>2.5, *P*<0.01, *d*_av_>0.43). These effects, however, dissipated after overnight consolidation after which no differences were found between Think and NoThink conditions in the bilateral hippocampus or amygdala (both *t*(17)<1.3, *P*>0.2). Further prediction analysis based on machine learning algorithms (see Methods section) revealed that hippocampal activity during the NoThink trials was negatively predictive of the suppression-induced forgetting score for newly acquired memories (*r*_(predicted, observed)_=−0.55, *P*=0.028) but not for the overnight condition (*r*_(predicted, observed)_=−0.21, *P*=0.65).

To further investigate neural systems underlying suppression-induced voluntary (intentional) forgetting (that is, NoThink trials later forgotten in post-scan test) and incidental forgetting (i.e., Think trials later forgotten in post-scan test) for overnight and newly acquired memories (Supplementary Fig. 6), we performed additional analysis only for NoThink and Think trials that were later forgotten in the 30 min and 24 h conditions (see Methods section for details). We artificially matched the number of trials between conditions and excluded four participants for their lack of forgotten trials. Again, we observed similar Suppression-by-Time interaction effects in the right DLPFC, the hippocampus and amygdala. Follow-up paired *t*-tests revealed higher activation in the right DLPFC for intentional relative to incidental forgetting for overnight memories (*t*(13)=3.36, *P*=0.01, *d*_av_=0.43), but not for newly acquired memories (*t*(13)=−0.38, *P*=0.71; Supplementary Fig. 7a). The differences in DLPFC engagement involved in intentional relative to incidental forgetting were higher for overnight memories than newly acquired ones (*t*(13)=3.02, *P*=0.016, *d*_av_=0.41; Supplementary Fig. 7b). Interestingly, we observed the opposite pattern in the hippocampus and amygdala, with significant decreased activation during intentional forgetting, relative to incidental forgetting of newly acquired memories (both *t*(13)<–2.5, *P*<0.04, *d*_av_>0.30), but not for overnight memories (both *t*(13)<1, *P*>0.35; Supplementary Fig. 7c,d). Taken together, these results indicate higher DLPFC engagement and less concomitant hippocampal and amygdala disengagement in suppression of overnight-consolidated aversive memories, even when considering only intentional forgetting.

### Distinct hippocampal–prefrontal pathways after consolidation

**Figure 4:**
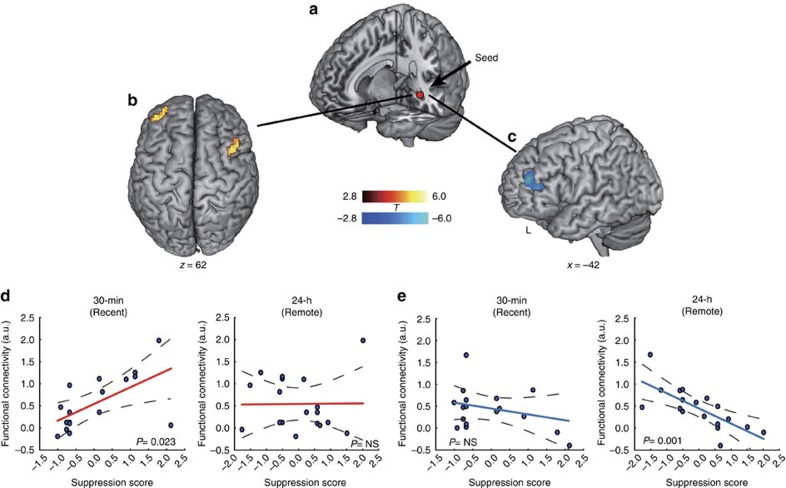
Distinct hippocampal-prefrontal functional pathways after consolidation. (**a**) The left hippocampal seed (MNI, peak at −33, −33 and −6) used in gPPI analysis of task-based functional connectivity during memory suppression. (**b**,**d**) Increased hippocampal functional connectivity with the left and right DLPFC was positively associated with more effective suppression for newly acquired memories (cluster in hot), but not after overnight consolidation. (**c**,**e**) Attenuated hippocampal functional connectivity with the left DLPFC was predictive of more effective suppression of overnight consolidated memories (cluster in blue), but not for newly acquired ones. Dotted lines indicate 95% confidence intervals and solid line indicates the best linear fit. MNI, Montreal Neurological Institute coordinate system.

To investigate how the hippocampal memory and prefrontal inhibitory control systems functionally coordinate to carry out suppression of aversive memories after overnight consolidation, we conducted a psychophysiological interaction (PPI) analysis (see Methods section) to identify functional coupling of the hippocampus with every other voxel of the brain, with a particular focus on the prefrontal inhibitory systems (Fig. 4a). This analysis revealed significant interaction effects in the DLPFC, the inferior frontal gyrus, and other regions (Supplementary Table 4). Further analysis revealed significantly lower hippocampal functional coupling with these regions in the suppression of aversive memories after overnight consolidation compared with the newly acquired condition (all *t*(17)>2.87, *P*<0.012, *d*_av_>0.45). The opposite pattern of results was observed in suppression of newly acquired aversive memories. Notably, increased hippocampal functional coupling with the bilateral DLPFC was predictive of more effective suppression of newly acquired memories (*r*_(predicted, observed)_=0.47, *P*=0.023; Fig. 4b,d). In contrast, decreased hippocampal functional coupling with the left DLPFC was predictive of more effective suppression of overnight consolidated memories (*r*_(predicted, observed)_=−0.70, *P*=0.001; Fig. 4c,e). In addition, we also performed hippocampal-seeded functional connectivity analysis only for forgotten trials by taking memory status into account. This analysis revealed a similar pattern of Suppression-by-Time interaction in the DLPFC (Supplementary Fig. 8). Altogether, these results indicate distinct hippocampal-prefrontal functional connectivity involved in suppression of overnight and newly acquired memories

### Distinct representational patterns after consolidation

**Figure 5:**
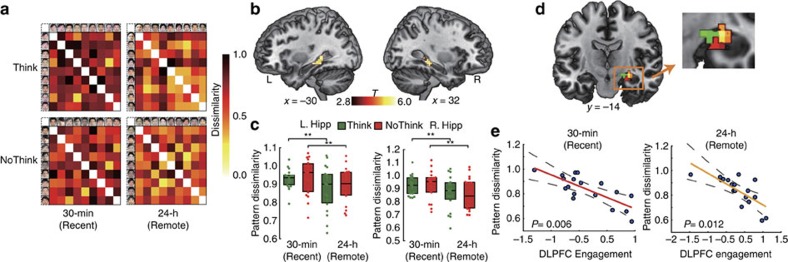
Decreased hippocampal pattern dissimilarity following consolidation. (**a**) 9 × 9 correlation matrix representing item-by-item pattern dissimilarity in the hippocampus for four separate experimental conditions. Each box corresponds to the specific item cue which is shown for visualization purposes. (**b**) Increased pattern dissimilarity in the bilateral hippocampus (MNI, left: peak at −33, −27 and −12; right: peak at 33, −36 and 0) for suppression of newly acquired compared with consolidated aversive memories, which is derived from inter-item multivoxel pattern dissimilarity analysis on the whole-brain level using a searchlight method. (**c**) Box plots reveal median (black line) multivoxel pattern dissimilarity in the bilateral hippocampus. Dots represent pattern dissimilarity for each participant. Error bars represent s.e.m. (**d**,**e**) Pattern dissimilarity in the anterior hippocampus was negatively predictive of DLPFC engagement in suppression of both newly acquired (cluster in hot) and consolidated (cluster in red) aversive memories. The cluster in green represents the overlapping area. Dashed lines indicate 95% confidence intervals, and solid lines indicate the best linear fit. *P<0.05; **P<0.01; Colour bar represents *T* values; a.u., arbitrary units; L, left; MNI, Montreal Neurological Institute coordinate system; R, right.

To further our understanding of how aversive memories become resistant to voluntary suppression after overnight consolidation, we investigated multivoxel activity patterns associated with individual aversive memories in newly acquired and overnight consolidation conditions. We implemented a multivariate pattern analysis that provides a measure of neural pattern dissimilarity, by examining inter-item correlational dissimilarity of multivoxel activity patterns within each condition. This analysis revealed a significant main effect of Time in the anatomically defined hippocampus (*F*(1, 17)=11.2, *P*=0.004, 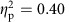; Fig. 5a), with lower multivoxel pattern dissimilarity (that is, greater pattern similarity) for aversive memories after overnight consolidation relative to the newly acquired condition for both recall (*t*(17)=2.54, *P*=0.021, *d*_av_=0.43) and suppression (*t*(17)=2.42, *P*=0.03, *d*_av_=0.42). Further analysis (see more details in the Supplementary Methods) revealed that the hippocampal representational dissimilarity was higher for intentional forgetting (NoThink trials later forgotten, NTf) versus incidental forgetting (Think trials later forgotten, Tf) of newly acquired aversive memories, but not for that of overnight aversive memories (Supplementary Fig. 9).

Using the searchlight algorithm^23^, we also performed a whole-brain exploratory analysis to identify changes in neural pattern dissimilarity associated with individual aversive memories after overnight consolidation. This analysis revealed significant clusters in the left and right hippocampus (peak at (−33, −27, −12), and (33, −36, 0) in MNI coordinates; Supplementary Table 5) that showed more generalized multivoxel activation patterns for aversive memories after overnight consolidation (Fig. 5b,c). Importantly, the hippocampal pattern dissimilarity was negatively predictive of the right DLPFC engagement in memory suppression for both newly acquired (*r*_(predicted, observed)_=−0.74, *P*=0.006) and overnight (*r*_(predicted, observed)_=−0.67, *P*=0.012) memories (Fig. 5d,e). There was no difference in the prediction slopes across newly acquired or overnight memories (*P*=0.89).

**Figure 6:**
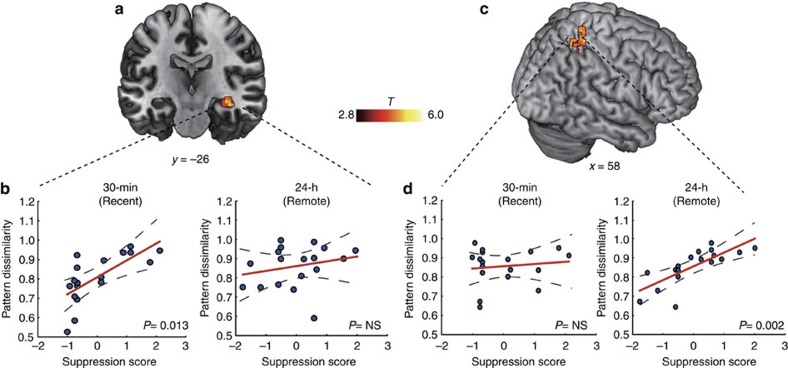
Distinct multivoxel representational patterns following consolidation. (**a**,**b**) Pattern dissimilarity in the hippocampus was positively associated with more effective suppression of newly acquired aversive memories, but not after overnight consolidation. (**c**,**d**) Pattern dissimilarity in the lateral parietal cortex was positively associated with more effective suppression of aversive memories after overnight consolidation, but not in the newly acquired condition. Dotted lines indicate 95% confidence intervals and red solid lines indicate the best linear fit. MNI, Montreal Neurological Institute coordinate system.

Critically, further analyses revealed that higher multivoxel pattern dissimilarity in the hippocampus was predictive of higher suppression-induced forgetting for newly acquired memories (*r*_(predicted, observed)_=0.66, *P*=0.013; Fig. 6a,b), whereas higher pattern dissimilarity in the neocortex (that is, LPC) was predictive of more effective suppression of overnight consolidated memories (*r*_(predicted, observed)_=0.69, *P*=0.002; Fig. 6c,d). Altogether, converging results from both ROI and whole brain analyses indicate neural activity patterns associated with individual aversive memories become more generalized (ie, less separable) in the hippocampus after overnight consolidation. Specially, distinct fine-tuned neural activity patterns in the hippocampus and the neocortex are predictive of suppression-induced forgetting of newly acquired and consolidated aversive memories, respectively.

## Discussion

We investigated physiological, behavioural and neural substrates underlying the effects of overnight consolidation on suppression of aversive memories. Behaviourally, the insertion of a 24-h delay made aversive memories become more resistant to suppression, with less suppression-induced forgetting and enduring emotional reactivity. These results were replicated in an independent cohort of 25 participants. Overnight memories appeared less dependent on the hippocampus and more dependent on the neocortex, a result most likely due to overnight consolidation. Suppression of these consolidated memories was associated with higher prefrontal engagement but less concomitant hippocampal and amygdala disengagement, with distinct hippocampal functional connectivity with prefrontal executive systems when compared with newly acquired memories. These effects were paralleled by a shift away from hippocampal-dependent representational patterns to distributed neocortical representational patterns following overnight consolidation. Our findings suggest that overnight consolidation promotes the assimilation of newly acquired memories into more distributed neocortical regions, making memories more resistant to suppression through prefrontal-hippocampal inhibitory pathways.

Beyond previous studies focusing on newly acquired memories^4^,^11^, our behavioural and SCR data converge on the notion that overnight consolidation makes aversive memories more resistant to suppression. For newly acquired memories, we replicated findings from many previous studies by showing prominent suppression-induced forgetting for the NoThink trials compared with the baseline trials^3^,^11^,^18^, which is in conjunction with significant reduction in SCR. These behavioural and physiological effects, however, were less pronounced after overnight consolidation, suggesting less effective voluntary suppression of aversive memories and associated emotional reactivity. Notably, our behavioural and physiological results were reproduced by an independent cohort of 25 participants, indicating the reliability and robustness of the observed effects. In addition, evidence from our behavioural control experiment with neutral stimuli provides similar effect in suppression of neutral memories after overnight consolidation, implying a general role of consolidation process on memory suppression irrespective of emotional specificity. From a broader perspective, our behavioural effects are reminiscent of results from recent studies indicating the reduced efficiency of voluntary suppression of established autobiographical memories^30^,^31^. This reduction is thought to result from memory consolidation processes^32^,^33^, including rapid eye movement sleep^34^. It is thus conceivable that overnight sleep may contribute to our observed effects of consolidation on aversive memories.

In conjunction with the above behavioural and SCR results, multiple aspects of our neuroimaging data from brain activation, functional connectivity and neural representation patterns consistently suggest that overnight consolidation promotes the assimilation of newly acquired memories into more distributed neocortical representations, thus making these memories more resistant to suppression. First, as predicted by classical consolidation models^1^,^15^, we observed that retrieval of newly acquired memories was associated with relatively greater hippocampal engagement, while recall of memories after consolidation was associated with greater engagement in the neocortical regions such as the LPC and angular gyrus. Similar hippocampal–neocortical functional reorganization involving consolidation of newly learned materials has been demonstrated in previous studies in humans and laboratory animals^8^,^22^,^35^. After consolidation, these memories may become assimilated into the neocortex and develop into more stable and less hippocampus-dependent representation through strengthening cortical-cortical connections^7^,^9^,^36^. In other words, overnight consolidation leads to large-scale functional reorganization of the MTL–neocortical networks involved in the transformation of aversive memories into more stable representations.

Second, suppression of aversive memories after consolidation appears to associate with increased prefrontal engagement but less concomitant hippocampal and amygdala disengagement when compared with newly acquired memories. Numerous studies involving the suppression of newly acquired materials have found increased engagement of the right DLPFC along with disengagement of the MTL regions^4^,^5^,^11^. We found a similar pattern in the suppression of newly acquired aversive memories. Although some regions involved in memory suppression seem to partially overlap with the default mode network (DMN), they differ substantially from the core DMN nodes when compared with the template derived from a large-scale meta-analysis of over 10 000 fMRI studies^37^. In fact, we observed significantly higher activation in the DLPFC and concomitant lower hippocampal activation during NoThink compared with Think trials. This is contradictory to the classic DMN hypothesis which predicts higher activity in DMN but lower activity in the central executive network such as DLPFC^38^. Consistent with findings from previous studies^5^,^12^, we believe that intentional suppression-induced forgetting involves the active and effortful inhibition of retrieval and access to memories^5^,^12^, rather than disengagement from the task.

More importantly, we observed a significant increase in right DLPFC activation during the suppression of aversive memories after overnight consolidation when compared with newly acquired memories. It appears that the right DLPFC is critical for inhibitory control over the hippocampal memory system in voluntary suppression processing^4^,^11^,^12^,^18^. The increased right DLPFC activation here may reflect more cognitive effort needed to suppress consolidated relative to recently acquired memories. This is in line with Anderson's executive control hypothesis, suggesting a U-shaped relationship between the levels of activation of the unwanted memory and the engagement of cognitive control^39^. In the present study, consolidated memories that became distributed in the neocortical network were more difficult to suppress, and thus required higher prefrontal inhibitory control than more recently acquired memories.

Furthermore, we observed less pronounced hippocampal and amygdala disengagement after overnight consolidation than for newly acquired memories. Previous studies using memory suppression tasks have demonstrated that memory suppression is associated with reduced activity in brain areas critical for episodic recollection (for example, hippocampus)^11^,^18^ and emotional memory (for example, amygdala)^4^. Our prediction analyses based on machine learning algorithms confirmed that the hippocampal disengagement is predictive of suppression-induced forgetting only in the newly acquired condition. Our observed maintenance of hippocampal and amygdala activity during suppression of consolidated aversive memories may reflect less effective prefrontal inhibitory control over the hippocampus and amygdala after overnight consolidation. This interpretation is consistent with less suppression-induced forgetting and less pronounced SCR reduction in the overnight consolidation condition.

It is worth noting that, even when only considering suppression-induced intentional forgetting versus incidental forgetting trials, we observed a very similar pattern of increased prefrontal engagement and less concomitant hippocampal and amygdala disengagement in suppression of overnight memories compared with newly acquired memories. This provides strong evidence that our observed effects truly reflect successful suppression-induced forgetting rather than the attempt to suppress. Although we cannot fully rule out potential confounds related to differences in general memory performance, memory decay on time, and interference between overnight and newly acquired memories (*cf*. Hulbert and Norman^40^), we undertook several steps to mitigate these confounding factors. For instance, we opted for an experimental design with specific baseline conditions separately for newly acquired and overnight consolidated memories. We then restricted our analyses for SCR and neuroimaging data by artificially matching the number of trials between these two conditions while taking subsequent memory status (remembered versus forgotten) into account. Together, converging evidence from behavioural, physiological and brain activation suggests that overnight consolidation makes aversive memories more resistant to suppression, which requires more prefrontal inhibitory control over hippocampal memory and amygdala emotional systems.

Third, suppression of overnight memories engages distinct hippocampal–prefrontal functional coupling processes and is associated with a shift away from hippocampal-dependent memory to more distributed neocortical representation patterns. Coordinated functional interactions between prefrontal executive and hippocampal memory systems are known to play a central role in memory suppression. Voluntary suppression of a memory is believed to involve prefrontal inhibitory control of the hippocampus where individual memories are processed^18^. Indeed, we observed increased hippocampal functional coupling with the right DLPFC during suppression of recently acquired memories. Analysis of forgotten trials further confirmed higher functional coupling between hippocampus and right DLPFC in the successful voluntary forgetting (that is, intentional versus incidental forgetting) of overnight memories. Interestingly, higher hippocampal-prefrontal functional coupling was predictive of greater suppression-induced forgetting of newly acquired memories. The opposite pattern of results was, however, observed in suppression of memories after overnight consolidation. These findings suggest that voluntary suppression through prefrontal inhibitory control over hippocampal memory circuitry is less effective and a distinct mechanism may be engaged in suppression of consolidated memories.

Moreover, results from multivoxel pattern similarity analysis revealed that individual aversive memories became more similar (that is, less discrete) following overnight consolidation in terms of inter-item multivoxel activity patterns (that is, less dissimilar to each other) in the hippocampus. These results provide evidence that individual memories encoded by the hippocampus may develop into less separable representations after overnight consolidation. Pattern separation is considered a hallmark of episodic memory, which allows numerous experiences to be differentiated^41^. The hippocampus is believed to play a central role in pattern separation by encoding disparate aspects of episodic memories as distinct non-overlapping representations to prevent interference across different memories^42^,^43^. Our observed increase in inter-item multivoxel pattern similarity in the hippocampus after overnight consolidation supports the notion that individual aversive memories become less discrete over time^44^. It is thus conceivable that the less efficient suppression of aversive memories found after overnight consolidation might be the result of less discrete neural representations of these memories, which make them less susceptible (thus more resistant) to prefrontal–hippocampal inhibitory processes as a result of a shift to distributed neocortical representations after overnight consolidation. In support of this idea we observed that higher multivoxel pattern dissimilarity in the hippocampus selectively predicted more effective suppression of newly acquired memories, but higher pattern dissimilarity in the LPC predicted more effective suppression of consolidated memories. In addition, we found that higher multivoxel pattern similarity in the hippocampus was associated with higher DLPFC engagement in memory suppression, suggesting increased prefrontal inhibitory efforts were required to suppress less discrete memory representations. This also explains our observed higher DLPFC activation in suppressing aversive memories after consolidation.

Taken together, our findings highlight the interaction between memory consolidation and suppression, and invite consideration of their possible clinical significance. Aberrant memory consolidation has been implicated in cognitive models of PTSD, and sleep is believed to play a vital role in this process^6^,^8^. These models posit that the emotional intensity of a traumatic memory is reduced during sleep through an active decoupling process whereby the emotional arousal is reduced and the non-emotional memory content is consolidated. If this process is prevented, for instance by disturbed sleep, the emotional ‘charge' of a traumatic memory remains, resulting in an inability to forget the trauma which is the hallmark of PTSD^8^. Related cognitive models of PTSD have led to promising therapeutic approaches^10^,^34^,^45^. For example, sleep deprivation immediately after traumatic experiences may prevent traumatic memories from being consolidated into stabilized representations and thus provide the opportunity to block the formation of traumatic memories^46^. In addition, it is also possible that our observed effects might simply be dependent on time rather than overnight sleep, or both. Our current design cannot separate general time- from sleep-dependent effects on memory consolidation. This possibility should be considered, since previous studies have showed that arousal-mediated consolidation effects are dependent on time, not sleep^47^ (but see Wilhelm *et al*.^48^). Future studies including a wake control group are needed to disentangle these effects.

In conclusion, our study demonstrates that suppression of consolidated aversive memories, compared with newly acquired memories, is characterized by less suppression-induced forgetting in conjunction with enduring emotional reactivity, which is associated with a higher prefrontal inhibitory control over hippocampal memory and amygdala emotional systems and a shift away from hippocampal-dependent representational patterns to distributed neocortical representational patterns. Our findings point towards a neurobiological model through which overnight consolidation assimilates aversive memories into more distributed neocortical representations, and makes these memories more resistant to suppression through the prefrontal–hippocampal inhibitory pathway. Our study underlines the importance of memory consolidation in understanding the resistance to suppression of emotional memories, which is a cardinal feature of affective disorders.

## Methods

### Participants

Twenty-one young healthy, male, right-handed college students (mean age±s.d., 21.6±0.81 years ranged from 20 to 24) with normal or corrected-to-normal vision participated in this study. Participants followed regular daily work-rest schedule, and reported no sleep-related problems during the last two months^49^. All participants reported good quality sleep during the night after training on day 1, with 7–9 h sleep time (mean±s.d.=7.35±0.37). Informed written consent was obtained from all participants before the experiment, and the study protocol was approved by the Institutional Review Board for Human Subjects at Beijing Normal University. Data from three participants were excluded from fMRI analyses due to excessive head movement during scanning. Note that another independent cohort of 25 participants (age range from 20 to 24 years old) was recruited for a parallel study using the same paradigm to examine the reproducibility of our behavioural and SCR findings. An additional behavioural control experiment with neutral stimuli was conducted in another independent 30 participants (age range from 20 to 24) to examine whether the observed effects of overnight consolidation on aversive memories were generalizable to neutral memories.

### General procedure

The whole experiment consisted of three phases: memory acquisition, memory suppression, and memory testing (Fig. 1). The memory acquisition phase comprised two sessions on day 1 and day 2, which occurred about 24 h (that is, training on day 1) and 30 min (that is, training on day 2) before fMRI scanning. On day 1, participants were extensively trained to learn and remember a set of 26 face-aversive picture associations. On day 2, they returned and were trained to remember another set of 26 face-picture associations. About 30 minutes after the training on day 2, participants underwent the scanning during which they were instructed to perform the memory suppression task using ‘Think/NoThink' paradigm^4^ in conjunction with concurrent recording of SCRs. Following that, participants performed a post-scan memory test for face-picture associations to assess their subsequent memory performance and effectiveness of memory suppression.

### Stimuli

Fifty-two face-aversive picture pairs were used in the present study. Fifty-two faces (26 males and 26 females) were carefully selected from 100 colour photographs of Chinese individuals unknown to the participants^50^. To standardize the stimuli and minimize potential confounding factors, faces were selected under the following criteria suggested by previous studies^51^,^52^: direct gaze contact, no strong emotional facial expression (which was rated as having a neutral expression in a pilot study on separate 9-point scales: 1=‘extremely sad' or ‘not arousing at all', 9=‘extremely happy' or ‘extremely arousing', with mean valence=5.16±0.53 and mean arousal=5.03±0.43), no headdress, no glasses, no beard, etc. There was no significant difference in terms of arousal, valence, attractiveness, and trustworthiness between male and female faces (all *P*>0.05). Subsequent analysis also revealed no difference in memory accuracy between male and female faces (*t*(17)=−0.88, *P*=0.39). Fifty-two aversive pictures from the International Affective Picture Series^53^ were carefully selected as having minimal relatedness in content with each other as possible, with a highly negative level of emotional valence (mean valence=2.37±0.69) and a high level of negative arousal (mean arousal=7.89±0.55) as measured on 9-point scales. Faces and aversive pictures were randomly paired across participants to create 52 face-picture associations. Associations were randomly assigned to day 1 and day 2. For behavioural control experiment (testing for neutral memories), 52 neutral pictures were chosen from International Affective Picture Series and online resources. They were carefully matched on complexity, luminance and contrasts with the aversive pictures, and have modest level of emotional valence (*M*=5.32±0.47) and low level of arousal (*M*=2.52±0.36) based on ratings from an independent sample.

### Memory acquisition

During the acquisition phase, participants performed a training session on day 1 and another on day 2 outside the scanner, which occurred 24 h and 30 min before the Think/NoThink task respectively. We carefully controlled the time interval between 30 min and 24 h on day 1 and day 2 to minimize variability across participants^54^. We thus restricted the first training phase to start at 16:00 hours on day 1, the scanning during ‘Think/NoThink' phase to 16:00–17:00 hours on day 2. The day 2 training started a half hour before scanning. In each training session, participants were trained to memorize 26 face-aversive picture pairs using multiple study–recall cycles. In each study–recall cycle, each association was presented for 4 s, and participants were encouraged to remember the association in detail. After presentation of all associations, participants were then shown a face and asked to recall details of the corresponding associated picture. This study–recall cycle was repeated 3–5 times until each participant could recall correctly all 26 face-picture associations. For each association, we required the participants to give detailed descriptions about the associated images when faces were presented as cues. The participants were asked to give enough details of that image to enable that it to be uniquely identified. This procedure was used to insure that participants formed vivid episodic memories for all associations rather than vague impressions based on familiarity^4^,^55^,^56^. Participants who required more than five training cycles to meet the criterion of 100% accuracy were excluded from the experiment. Note that we restricted the training phase within 3–5 cycles to avoid extensive training which may result in memories too strong to suppression (3,5). 

### TNT phase

In the TNT Phase, participants underwent fMRI with concurrent recording of SCRs while performing the TNT task for face-picture associations acquired on day 1 and day 2. Each trial started with presentation of a face for 4 s, and was followed an inter-trial interval with a fixation cross for 2–6 s (average duration=4 s). The ‘Think' and ‘NoThink' trials were pseudo-randomized across participants and interleaved by a fixation period. The instructional cues (i.e., green and red rectangles) indicated ‘Think' and ‘NoThink' trials respectively, which appeared simultaneously with the face presentation. The presentation of only faces in this phase ensured that participants manipulated associated memories of the target picture^4^,^57^. When seeing an instructional cue ‘Think' (green rectangle), participants were required to recall and think of the previously learned picture, and when seeing an instructional cue ‘NoThink' (red rectangle), they were instructed to not let the associated picture enter consciousness. After the presentation of a face, a fixation was presented during an inter-trial interval which served as a low level baseline for the experimental trials^4^. The total duration of the task was 19.2 min with 144 trials in total with 36 trials in each condition. Participants were shown 36 out of 52 faces in total, with half of them acquired on day 1 around 24 h before the task (that is, overnight condition) and the other half acquired on day 2 about 30 min before the task (that is, newly acquired condition). Cues for baseline pairs were not presented in this phase. Half of these faces were randomly assigned to either Think (that is, ‘T) or NoThink (that is, ‘NT') condition, resulting in four experimental conditions in a 2-by-2 full factorial design (that is, memory suppression: NoThink versus Think, acquisition time: 30 min versus 24 h) with 9 faces per condition. Each face repeated four times, resulted in 144 trials in total. The remaining 16 faces (half of them acquired 24 h before the task) were not included in the TNT task, served as a behavioural baseline. Before the fMRI scanning, participants were trained twice using 10 trials that were not used in the actual experiment. Participants were explicitly instructed to directly suppress unwanted aversive memories by attempting to exclude a memory from awareness, rather than occupying awareness with another competing thought (i.e., thought substitution)^13^.

### Post-scan memory test

In the testing phase (Fig. 1d), memory performance for face-picture associations was assessed by a cued-recall task, in which all 52 faces (learned on day 1 and day 2) were included. Each trial started with a face as a cue, and participants were encouraged to recall details of the associated picture. On each trial, participants were given a maximum of 30 s to verbally describe the associated pictures in as much detail as possible. The description was only scored as correct (that is, remembered) if it included enough details for the specific scene to be uniquely identified^57^. Incorrect or vague descriptions were treated as forgotten. Three raters who were blinded to the experiment reviewed participants' answers independently. The final test scores were cross-validated by three raters. A final judgment on each item was only made when a consensus was reached among the three raters. If there was any disagreement, the three raters discussed them and made a collective decision. In the TNT phase, the baseline items were not present in TNT task and thus with less presentation times. This has been widely used to provide a baseline measure of memory performance that is not directly affected by ‘Think' or ‘NoThink' manipulation^3^,^4^,^11^.

### Behavioural data analysis

Memory accuracy was submitted to a 2-by-3 repeated-measures ANOVA with memory suppression (Memory: Baseline versus versus Think versus NoThink) and acquisition time (Time: 30 min versus 24 h) as within-subject factors. Similar to Levy and Anderson^18^, we also computed the suppression score for overnight (24 h) and newly acquired (30-min) memories by subtracting corresponding memory accuracy of ‘NoThink' items from their respective ‘Baseline' items to provide a measure of suppression efficiency. Individual participant's suppression score was then *Z*-normalized for further brain-behavioural prediction analyses.

### SCR recording

SCR was recorded simultaneously with fMRI scanning using a Biopac MP 150 System (Biopac, Inc., Goleta, CA). Two Ag/AgCl electrodes filled with isotonic electrolyte medium were attached to the center phalanges of the index and middle fingers of the left hand. The gain set to 5, the low pass filter set to 1.0 Hz, and the high pass filters set to DC^58^. Data were acquired at 200 samples per second. Before analysis, the data were transformed into microsiemens (μS) and square root transformed due to non-normality of the data distribution.

### SCR analysis

SCR data were analysed offline using Matlab R2014a (MathWorks, Natick, USA). First, data were temporally smoothed with a median filter (that is, 40 samples within a 200 ms window) to reduce scanner-induced noise^59^,^60^. We used Autonomate^61^ to analyse event-related SCRs, which has been proven effective in the context of event-related cognitive tasks^2^,^61^. In brief, the electrodermal data were segmented into event-related time windows based on face onset. The face-related SCR was located by identifying rises in the electrodermal data, which constituted the onset of an SCR. Responses that did not fit these criteria were scored as zero. Noisy segments of data (in which an implausible number of candidate SCRs were present) were excluded from further analysis (see more details in the Supplementary Methods). The resulting number of trials used for SCR analyses are reported in the Supplementary Table 6. SCR values were then identified as the maximum value within each time window. If multiple SCRs fell within the same window, the largest response was scored^61^. A 2-by-2 repeated-measures ANOVA with memory suppression (Think versus NoThink) and acquisition time (30 min versus 24 h) as within-subject factors was used to examine physiological changes as a function of 4 conditions for cognitive manipulations on aversive memories.

### Imaging acquisition

Whole-brain imaging data was collected on a Siemens TRIO 3-Tesla MR scanner in the National Key Laboratory of Cognitive Neuroscience and Learning at Beijing Normal University. Functional images were collected using an echo-planar imaging sequence (axial slices, 33; slice thickness, 4 mm; gap, 0.6 mm; TR, 2000, ms; TE, 30 ms; flip angle, 90°; voxel size, 3.1 × 3.1 × 4.0 mm; flip angle, 90°; FOV, 200 × 200 mm; and 580 volumes), while structural images were acquired through three-dimensional sagittal T1-weighted magnetization-prepared rapid gradient echo (192 slices; TR, 2530, ms; TE, 3.45 ms; slice thickness, 1 mm; voxel size, 1.0 × 1.0 × 1.0 mm^3^; flip angle, 7°; inversion time, 1100, ms; FOV, 256 × 256 mm).

### Imaging preprocessing

Brain imaging data was preprocessed using Statistical Parametric Mapping (SPM8; http://www.fil.ion.ucl.ac.uk/spm). The first 4 volumes of functional images were discarded for signal equilibrium and participants' adaptation to scanning noise. Remaining images were corrected for slice acquisition timing and realigned for head motion correction. Subsequently, functional images were co-registered to each participant's gray matter image segmented from corresponding high-resolution T1-weighted image, then spatially normalized into a common stereotactic Montreal Neurological Institute (MNI) space and resampled into 3-mm isotropic voxels. Finally, images were smoothed by an isotropic three-dimensional Gaussian kernel with 4 mm full-width at half-maximum. The data were statistically analysed under the framework of general linear models (GLM)^62^.

### Univariate GLM analysis

To assess transient neural activity associated with memory retrieval (that is, Think trials) and suppression (that is, NoThink trials) for newly acquired and overnight aversive memories, separate regressors of interest were modelled for four experimental conditions (see above) and convolved with the canonical hemodynamic response function (HRF) at the first level. In addition, each participant's motion parameters from the realignment procedure were included to regress out effects related to head movement-related variability. The analyses included high-pass filtering using a cutoff of 1/40 hz to remove high frequency noise^4^, global intensity normalization and corrections for serial correlations using a first-order autoregressive model (AR(1)). Relevant contrast parameter estimate images were initially generated at the individual-subject level, and then submitted to a 2 (Suppression) by 2 (Time) repeated-measures ANOVA for a second-level group analysis treating participants as a random variable. Significant clusters were identified from the group analysis, initially masked using a gray matter mask, and then determined using conservative and well-accepted statistical criteria—that is, a height threshold of *P*<0.01 and an extent threshold of *P*<0.05 with family-wise error corrections for multiple comparisons based on nonstationary suprathreshold cluster-size distributions computed using Monte Carlo simulations^63^.

To further investigate specific neural activity associated with suppression-induced voluntary or intentional forgetting (that is, NTf) and incidental forgetting (that is, Tf), we conducted an additional GLM analysis by including memory Status (forgotten versus remembered, or f versus r) as another variable of interest, similar to Anderson *et al*.^11^, together with Time (30 min versus 24 h) and Suppression (NoThink versus Think, or NT versus T). This analysis included eight regressors of interest (that is, NTf_30 min, NTr_30 min, Tf_30 min, Tr_30 min, NTf_24 h, NTr_24 h, Tf_24 h, and Tr_24 h). We used random sub-samplings from the 30 min conditions to artificially match the number of items in the 30 min and 24 h conditions being used for the first level individual analysis. This allowed us to compare neural activity in successful suppression trials between newly acquired and overnight memories with similar statistical power (that is, [NTf_24 h—Tf_24 h] versus [NTf_30 min—Tf_30 min]). Four participants were excluded from this analysis due to lack of at least one forgotten item in each condition. Relevant parameter contrasts were then submitted to 2 (Status: forgotten versus remembered) by 2 Time (Time) by 2 (Suppression) repeated-measures ANOVA for a second-level group analysis treating participants as a random variable. All other settings were same as the above GLM analysis.

To better characterize hippocampal and prefrontal engagement in memory suppression, we performed complementary ROI analyses separately for the left and right entire hippocampus and the middle frontal gyrus (referred to as DLPFC) anatomically defined using the WFU PickAtlas toolbox^64^. Parameter estimates (or β-weights) associated with conditions of interest were extracted from the above anatomically defined ROIs as well as significant clusters in the MTL and PFC regions at the individual level using MarsBar (http://marsbar.sourceforge.net/) and averaged across voxels within each ROI, then plotted in bar graphs for visualization purposes only.

### Task-dependent functional connectivity analysis

We examined hippocampus-based functional connectivity changes via PPI analysis^65^. The hippocampal seed was separately defined as a 4-mm sphere centered at the local peak of corresponding clusters showing significant interaction effects between Suppression and Time in the univariate GLM analysis. To accommodate more than two experimental conditions within same model, we employed a generalized form of task-dependent PPI (gPPI)^66^ The physiological activity of given hippocampal seed region was computed as the mean time series of all voxels. They were then deconvolved to estimate neural activity. Next, four PPI regressors, corresponding to each task regressor from the individual level were obtained by multiplying the estimated neuronal activity from the seed region with a vector coding for effects of each condition, forming four psychophysiological interaction vectors. They were further convolved with a canonical HRF to form four PPI regressors of interest. Task-related activations were also included in this GLM to remove out the effects of common driving inputs on brain connectivity.

Contrast images corresponding to PPI effects at the individual-subject level were then submitted to a 2 (Suppression) by 2 (Time) repeated-measures ANOVA for a second-level group analysis. Similar to the univariate GLM analysis above, significant clusters were initially masked by a gray matter mask, and then determined using a height threshold of *P*<0.01 and an extent threshold of *P*<0.05 with family-wise error correction for multiple comparisons based on nonstationary suprathreshold cluster-size distributions^63^.

To investigate brain functional connectivity patterns associated with intentional forgetting and incidental forgetting between 30-min and 24-h conditions, we conducted an additional hippocampal-seeded PPI analysis by taking memory status into account, with a particular focus on NoThink trials that were later forgotten in the post-scan testing phase (ie, [NTf_24 h—Tf_24 h] versus [NTf_30 min—Tf_30 min]). The seed voxels for the connectivity analysis were chosen around the peak coordinate of the hippocampal cluster with a 4-mm sphere of voxels identified from the additional GLM analysis while taking memory status into account. Other settings were same as the above PPI analysis.

### Prediction analysis

We employed a machine-learning approach with balanced fourfold cross-validation to mitigate shortcomings of conventional regression models and test for generalizability of the established relationship to out-of-sample individual subjects^67^. For example, we entered memory suppression scores for each individual as dependent variable, and hippocampal activation as independent variable. Then, we estimated *r*_(predicted, observed)_ to measure how well the hippocampal activations predict the memory suppression scores using a balanced fourfold cross-validation procedure. Data were divided into four folds, and a linear regression model was built using three folds, leaving one fold out. A final *r*_(predicted, observed)_ was computed based on the average of four repetition of this procedure. Finally, we used a nonparametric testing approach to test for the statistical significance of the model by generating 1,000 surrogate data sets under the null hypothesis of *r*_(predicted, observed)_ (ref. 68). The statistical significance (*P* value) of the model was determined by measuring the percentage of generated surrogate data that are greater than the *r*_(predicted, observed)._

### Multivoxel pattern dissimilarity analysis

To assess multivoxel pattern dissimilarity associated with newly acquired and overnight aversive memories, we modelled each item (collapsing across four repetitions) as a separate regressor, convolved with a canonical HRF implemented in SPM8. This resulted in 36 regressors in total and 9 regressors for each condition. Contrast images for each item versus fixation, generated at the individual level analysis within each condition were then submitted to subsequent inter-item multivariate pattern dissimilarity analysis for the hippocampal ROIs as well as for the whole brain.

### ROI-based pattern dissimilarity analysis

For each of four experimental conditions, we extracted voxel-wise brain activation estimates for each item within the same condition from the defined ROIs, and reshaped them into a single dimensional vector for each ROI. Pairwise correlations were then computed among distributed voxels of each ROI, resulting in *N* × (*N*−1)/2 pairwise correlation coefficients, with *N* representing the number of items in each condition. The dissimilarity score was determined by Fisher's *Z* transformation of 1 minus the correlation coefficient, separately for each participant^24^,^25^,^26^. The data were then submitted to a 2-by-2 repeated-measures ANOVA with Suppression and Time as within-subject factors for the second-level analysis to investigate differences in pattern dissimilarity between retrieval and suppression of newly acquired and overnight-consolidated aversive memories.

### Whole-brain pattern dissimilarity analysis

We further implemented a searchlight method to measure inter-item multivoxel pattern dissimilarity at the whole brain level^23^,^24^, using a 6-mm spherical region of interest^69^. As with the ROI-based analysis, we computed the inter-item multivoxel pattern similarity for each condition within each searchlight. The analysis was then repeated for a searchlight centered on every voxel in the brain. Searchlight maps for all four conditions were then entered into a 2-by-2 ANOVA with Suppression and Time as within-subject factors on the second level group analysis to determine changes in pattern dissimilarity between retrieval and suppression of newly acquired and overnight-consolidated aversive memories. Significant clusters were identified using a height threshold of *P*<0.01 and an extent threshold of *P*<0.05 with family-wise error corrections^63^.

To further examine the relationship between memory suppression and neural representation patterns, we performed separate regression analyses for the whole-brain pattern dissimilarity maps with suppression-induced forgetting scores in newly acquired or overnight consolidation condition as a covariate of interest. Parallel analyses were also conducted to examine the relationship between hippocampal pattern dissimilarity and DLPFC engagement in the suppression of either newly acquired or consolidated aversive memories, separately. The significant clusters were determined using the same criterion from the above GLM and gPPI analyses.

### Estimates of effect size and post-hoc statistical power

Effect sizes for ANOVAs are partial eta squared, referred to as 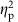. For paired *t*-tests, we calculated Cohen's *d* using the mean difference score as the numerator and the pooled s.d. from both repeated measures as the denominator^70^. This effect size is referred to in the text as *d*_av_, in which the ‘av' refers to the use of the average s.d. in the calculation. The post-hoc statistical power was calculated based on the given type I error rate (*α*=0.05), the corresponding sample size and effect size.

### Data availability

The data and codes that support the findings of this study are available from the corresponding author on request.

## Additional information

**How to cite this article:** Liu, Y. *et al*. Memory consolidation reconfigures neural pathways involved in the suppression of emotional memories. *Nat. Commun.* **7,** 13375 doi: 10.1038/ncomms13375 (2016).

**Publisher's note**: Springer Nature remains neutral with regard to jurisdictional claims in published maps and institutional affiliations.

## Supplementary Material

Supplementary Information Supplementary Figures 1 - 9, Supplementary Tables 1 - 6 and Supplementary Methods

## References

[b1] McGaugh J. L. Memory—a century of consolidation. Science 287, 248–251 (2000). 10634773 10.1126/science.287.5451.248

[b2] Dunsmoor J. E., Murty V. P., Davachi L. & Phelps E. A. Emotional learning selectively and retroactively strengthens memories for related events. Nature 520, 345–348 (2015). 25607357 10.1038/nature14106 PMC4432479

[b3] Anderson M. C. & Green C. Suppressing unwanted memories by executive control. Nature 410, 366–369 (2001). 11268212 10.1038/35066572

[b4] Depue B. E., Curran T. & Banich M. T. Prefrontal regions orchestrate suppression of emotional memories via a two-phase process. Science 317, 215–219 (2007). 17626877 10.1126/science.1139560

[b5] Anderson M. C. & Hanslmayr S. Neural mechanisms of motivated forgetting. Trends Cogn. Sci. 18, 279–292 (2014). 24747000 10.1016/j.tics.2014.03.002 PMC4045208

[b6] Catarino A., Küpper C. S., Werner-Seidler A., Dalgleish T. & Anderson M. C. Failing to forget inhibitory-control deficits compromise memory suppression in posttraumatic stress disorder. Psychol. Sci. 26, 604–616 (2015). 25847536 10.1177/0956797615569889 PMC4426138

[b7] Walker M. P. & Stickgold R. Sleep-dependent learning and memory consolidation. Neuron 44, 121–133 (2004). 15450165 10.1016/j.neuron.2004.08.031

[b8] Walker M. P. & van Der Helm E. Overnight therapy? The role of sleep in emotional brain processing. Psychol. Bull. 135, 731 (2009). 19702380 10.1037/a0016570 PMC2890316

[b9] Stickgold R. Sleep-dependent memory consolidation. Nature 437, 1272–1278 (2005). 16251952 10.1038/nature04286

[b10] Stickgold R. & Walker M. P. Sleep-dependent memory triage: evolving generalization through selective processing. Nat. Neurosci. 16, 139–145 (2013). 23354387 10.1038/nn.3303 PMC5826623

[b11] Anderson M. C. . Neural systems underlying the suppression of unwanted memories. Science 303, 232–235 (2004). 14716015 10.1126/science.1089504

[b12] Levy B. J. & Anderson M. C. Inhibitory processes and the control of memory retrieval. Trends Cogn. Sci. 6, 299–305 (2002). 12110363 10.1016/s1364-6613(02)01923-x

[b13] Benoit R. G. & Anderson M. C. Opposing mechanisms support the voluntary forgetting of unwanted memories. Neuron 76, 450–460 (2012). 23083745 10.1016/j.neuron.2012.07.025 PMC3480638

[b14] McGaugh J. L. The amygdala modulates the consolidation of memories of emotionally arousing experiences. Annu. Rev. Neurosci. 27, 1–28 (2004). 15217324 10.1146/annurev.neuro.27.070203.144157

[b15] Frankland P. W. & Bontempi B. The organization of recent and remote memories. Nat. Rev. Neurosci. 6, 119–130 (2005). 15685217 10.1038/nrn1607

[b16] Squire L. R. & Bayley P. J. The neuroscience of remote memory. Curr. Opin. Neurobiol. 17, 185–196 (2007). 17336513 10.1016/j.conb.2007.02.006 PMC2277361

[b17] Bayley P. J., Hopkins R. O. & Squire L. R. Successful recollection of remote autobiographical memories by amnesic patients with medial temporal lobe lesions. Neuron 38, 135–144 (2003). 12691671 10.1016/s0896-6273(03)00156-9

[b18] Levy B. J. & Anderson M. C. Purging of memories from conscious awareness tracked in the human brain. J. Neurosci. 32, 16785–16794 (2012). 23175832 10.1523/JNEUROSCI.2640-12.2012 PMC3544307

[b19] Gagnepain P., Henson R. N. & Anderson M. C. Suppressing unwanted memories reduces their unconscious influence via targeted cortical inhibition. Proc. Natl Acad. Sci. USA 111, E1310–E1319 (2014). 24639546 10.1073/pnas.1311468111 PMC3977236

[b20] Dudai Y. The neurobiology of consolidations, or, how stable is the engram? Annu. Rev. Psychol. 55, 51–86 (2004). 14744210 10.1146/annurev.psych.55.090902.142050

[b21] Shimizu E., Tang Y.-P., Rampon C. & Tsien J. Z. NMDA receptor-dependent synaptic reinforcement as a crucial process for memory consolidation. Science 290, 1170–1174 (2000). 11073458 10.1126/science.290.5494.1170

[b22] Takashima A. . Shift from hippocampal to neocortical centered retrieval network with consolidation. J. Neurosci. 29, 10087–10093 (2009). 19675242 10.1523/JNEUROSCI.0799-09.2009 PMC6664975

[b23] Kriegeskorte N., Goebel R. & Bandettini P. Information-based functional brain mapping. Proc. Natl Acad. Sci. USA 103, 3863–3868 (2006). 16537458 10.1073/pnas.0600244103 PMC1383651

[b24] Kriegeskorte N., Mur M. & Bandettini P. Representational similarity analysis–connecting the branches of systems neuroscience. Front. Syst. Neurosci. 2, 4 (2008). 19104670 10.3389/neuro.06.004.2008 PMC2605405

[b25] Kriegeskorte N. & Kievit R. A. Representational geometry: integrating cognition, computation, and the brain. Trends Cogn. Sci. 17, 401–412 (2013). 23876494 10.1016/j.tics.2013.06.007 PMC3730178

[b26] Haxby J. V., Connolly A. C. & Guntupalli J. S. Decoding neural representational spaces using multivariate pattern analysis. Annu. Rev. Neurosci. 37, 435–456 (2014). 25002277 10.1146/annurev-neuro-062012-170325

[b27] Ezzyat Y. & Davachi L. Similarity breeds proximity: pattern similarity within and across contexts is related to later mnemonic judgments of temporal proximity. Neuron 81, 1179–1189 (2014). 24607235 10.1016/j.neuron.2014.01.042 PMC3983791

[b28] Hsieh L.-T., Gruber M. J., Jenkins L. J. & Ranganath C. Hippocampal activity patterns carry information about objects in temporal context. Neuron 81, 1165–1178 (2014). 24607234 10.1016/j.neuron.2014.01.015 PMC3984944

[b29] Wimber M., Alink A., Charest I., Kriegeskorte N. & Anderson M. C. Retrieval induces adaptive forgetting of competing memories via cortical pattern suppression. Nat. Neurosci. 18, 582–589 (2015). 25774450 10.1038/nn.3973 PMC4394359

[b30] Stephens E., Braid A. & Hertel P. T. Suppression-induced reduction in the specificity of autobiographical memories. Clin. Psychol. Sci. 1, 163–169 (2013). 25161828 10.1177/2167702612467773 PMC4142200

[b31] Noreen S. & Macleod M. D. It's all in the detail: intentional forgetting of autobiographical memories using the autobiographical think/no-think task. J. Exp. Psychol. Learn. Mem. Cogn. 39, 375–393 (2013). 22686849 10.1037/a0028888

[b32] Noreen S. & MacLeod M. D. To think or not to think, that is the question: Individual differences in suppression and rebound effects in autobiographical memory. Acta Psychol. 145, 84–97 (2014). 10.1016/j.actpsy.2013.10.011 24309017

[b33] Fischer S., Diekelmann S. & Born J. Sleep's role in the processing of unwanted memories. J. Sleep Res. 20, 267–274 (2011). 20723021 10.1111/j.1365-2869.2010.00881.x

[b34] Siegel J. M. The REM sleep-memory consolidation hypothesis. Science 294, 1058–1063 (2001). 11691984 10.1126/science.1063049 PMC8760621

[b35] Takashima A. . Declarative memory consolidation in humans: a prospective functional magnetic resonance imaging study. Proc. Natl Acad. Sci. USA 103, 756–761 (2006). 16407110 10.1073/pnas.0507774103 PMC1334654

[b36] Wiltgen B. J., Brown R. A. M., Talton L. E. & Silva A. J. New circuits for old memories: the role of the neocortex in consolidation. Neuron 44, 101–108 (2004). 15450163 10.1016/j.neuron.2004.09.015

[b37] Yarkoni T., Poldrack R. A., Nichols T. E., Van Essen D. C. & Wager T. D. Large-scale automated synthesis of human functional neuroimaging data. Nat. Methods 8, 665–670 (2011). 21706013 10.1038/nmeth.1635 PMC3146590

[b38] Raichle M. E. The brain's default mode network. Annu. Rev. Neurosci. 38, 433–447 (2015). 25938726 10.1146/annurev-neuro-071013-014030

[b39] Anderson M. C. & Levy B. J. in *Successful* *Remembering and Successful Forgetting,* 107–132 (Psychology Press, 2010).

[b40] Hulbert J. & Norman K. Neural differentiation tracks improved recall of competing memories following interleaved study and retrieval practice. Cereb. Cortex 25, 3994–4008 (2015). 25477369 10.1093/cercor/bhu284 PMC4585527

[b41] Favila S. E., Chanales A. J. & Kuhl B. A. Experience-dependent hippocampal pattern differentiation prevents interference during subsequent learning. Nat. Commun. 7, 11066 (2016). 10.1038/ncomms11066 PMC4820837 27925613

[b42] Leutgeb J. K., Leutgeb S., Moser M.-B. & Moser E. I. Pattern separation in the dentate gyrus and CA3 of the hippocampus. Science 315, 961–966 (2007). 17303747 10.1126/science.1135801

[b43] Yassa M. A. & Stark C. E. L. Pattern separation in the hippocampus. Trends Neurosci. 34, 515–525 (2011). 21788086 10.1016/j.tins.2011.06.006 PMC3183227

[b44] Kheirbek M. A., Klemenhagen K. C., Sahay A. & Hen R. Neurogenesis and generalization: a new approach to stratify and treat anxiety disorders. Nat. Neurosci. 15, 1613–1620 (2012). 23187693 10.1038/nn.3262 PMC3638121

[b45] Germain A. Sleep disturbances as the hallmark of PTSD: where are we now? Am. J. Psychiatry 170, 372–382 (2013). 23223954 10.1176/appi.ajp.2012.12040432 PMC4197954

[b46] Kuriyama K., Soshi T. & Kim Y. Sleep deprivation facilitates extinction of implicit fear generalization and physiological response to fear. Biol. Psychiatry 68, 991–998 (2010). 20889142 10.1016/j.biopsych.2010.08.015

[b47] Park J. Effect of arousal and retention delay on memory: a meta-analysis. Psychol. Rep. 97, 339–355 (2005). 16342564 10.2466/pr0.97.2.339-355

[b48] Wilhelm I. . Sleep selectively enhances memory expected to be of future relevance. J. Neurosci. 31, 1563–1569 (2011). 21289163 10.1523/JNEUROSCI.3575-10.2011 PMC6623736

[b49] Tsai P.-S. . Psychometric evaluation of the Chinese version of the Pittsburgh Sleep Quality Index (CPSQI) in primary insomnia and control subjects. Qual. Life Res. 14, 1943–1952 (2005). 16155782 10.1007/s11136-005-4346-x

[b50] Chen C. . Suppression of aversive memories associates with changes in early and late stages of neurocognitive processing. Neuropsychologia 50, 2839–2848 (2012). 22917568 10.1016/j.neuropsychologia.2012.08.004

[b51] Sperling R. . Putting names to faces:: Successful encoding of associative memories activates the anterior hippocampal formation. NeuroImage 20, 1400–1410 (2003). 14568509 10.1016/S1053-8119(03)00391-4 PMC3230827

[b52] Qin S. . Probing the transformation of discontinuous associations into episodic memory: An event-related fMRI study. NeuroImage 38, 212–222 (2007). 17804259 10.1016/j.neuroimage.2007.07.020

[b53] Lang P. J., Bradley M. M. & Cuthbert B. N. in *International affective* *picture system (IAPS): Affective ratings of pictures and instruction manual* (NIMH, Center for the Study of Emotion & Attention, 2005).

[b54] Wessel I., Huntjens R. J. & Verwoerd J. R. Cognitive control and suppression of memories of an emotional film. J. Behav. Ther. Exp. Psychiatry 41, 83–89 (2010). 19896117 10.1016/j.jbtep.2009.10.005

[b55] Yonelinas A. P., Otten L. J., Shaw K. N. & Rugg M. D. Separating the brain regions involved in recollection and familiarity in recognition memory. J. Neurosci. 25, 3002–3008 (2005). 15772360 10.1523/JNEUROSCI.5295-04.2005 PMC6725129

[b56] Yonelinas A. P. & Ritchey M. The slow forgetting of emotional episodic memories: an emotional binding account. Trends Cogn. Sci. 19, 259–267 (2015). 25836045 10.1016/j.tics.2015.02.009 PMC4414918

[b57] Depue B. E., Banich M. T. & Curran T. Suppression of emotional and nonemotional content in memory effects of repetition on cognitive control. Psychol. Sci. 17, 441–447 (2006). 16683933 10.1111/j.1467-9280.2006.01725.x PMC2878760

[b58] Indovina I., Robbins T. W., Núñez-Elizalde A. O., Dunn B. D. & Bishop S. J. Fear-conditioning mechanisms associated with trait vulnerability to anxiety in humans. Neuron 69, 563–571 (2011). 21315265 10.1016/j.neuron.2010.12.034 PMC3047792

[b59] Kim K. H., Bang S. W. & Kim S. R. Emotion recognition system using short-term monitoring of physiological signals. Med. Biol. Eng. Comput. 42, 419–427 (2004). 15191089 10.1007/BF02344719

[b60] McMenamin B. W., Langeslag S. J. E., Sirbu M., Padmala S. & Pessoa L. Network organization unfolds over time during periods of anxious anticipation. J. Neurosci. 34, 11261–11273 (2014). 25143607 10.1523/JNEUROSCI.1579-14.2014 PMC4138337

[b61] Green S. R., Kragel P. A., Fecteau M. E. & LaBar K. S. Development and validation of an unsupervised scoring system (Autonomate) for skin conductance response analysis. Int. J. Psychophysiol. 91, 186–193 (2014). 24184342 10.1016/j.ijpsycho.2013.10.015 PMC3943713

[b62] Friston K. J. . Analysis of fMRI time-series revisited. NeuroImage 2, 45–53 (1995). 9343589 10.1006/nimg.1995.1007

[b63] Nichols T. & Hayasaka S. Controlling the familywise error rate in functional neuroimaging: a comparative review. Stat. Methods Med. Res. 12, 419–446 (2003). 14599004 10.1191/0962280203sm341ra

[b64] Maldjian J. A., Laurienti P. J., Kraft R. A. & Burdette J. H. An automated method for neuroanatomic and cytoarchitectonic atlas-based interrogation of fMRI data sets. NeuroImage 19, 1233–1239 (2003). 12880848 10.1016/s1053-8119(03)00169-1

[b65] Friston K. . Psychophysiological and modulatory interactions in neuroimaging. NeuroImage 6, 218–229 (1997). 9344826 10.1006/nimg.1997.0291

[b66] McLaren D. G., Ries M. L., Xu G. & Johnson S. C. A generalized form of context-dependent psychophysiological interactions (gPPI): a comparison to standard approaches. NeuroImage 61, 1277–1286 (2012). 22484411 10.1016/j.neuroimage.2012.03.068 PMC3376181

[b67] Dubois J. & Adolphs R. Building a Science of Individual Differences from fMRI. Trends Cogn. Sci. 20, 425–443 (2016). 27138646 10.1016/j.tics.2016.03.014 PMC4886721

[b68] Yarkoni T. & Westfall J. in *Choosing prediction over explanation in psychology: Lessons from machine learning* (Figshare, 2016). 10.1177/1745691617693393 PMC6603289 28841086

[b69] Etzel J. A., Zacks J. M. & Braver T. S. Searchlight analysis: promise, pitfalls, and potential. NeuroImage 78, 261–269 (2013). 23558106 10.1016/j.neuroimage.2013.03.041 PMC3988828

[b70] Lakens D. Calculating and reporting effect sizes to facilitate cumulative science: a practical primer for t-tests and ANOVAs. Front. Psychol. 4, 863 (2013). 24324449 10.3389/fpsyg.2013.00863 PMC3840331

