# Supplementary material for: Memory consolidation reconfigures neural pathways involved in the suppression of emotional memories
Source: Nat Commun. 2016 Nov 29;7:13375. doi: 10.1038/ncomms13375 (PMC5141344; doi:10.1038/ncomms13375)
Supplement: Supplementary Information — Supplementary Figures 1 - 9, Supplementary Tables 1 - 6 and Supplementary Methods [file ncomms13375-s1.pdf]

Supplemental Figures 1 - 9

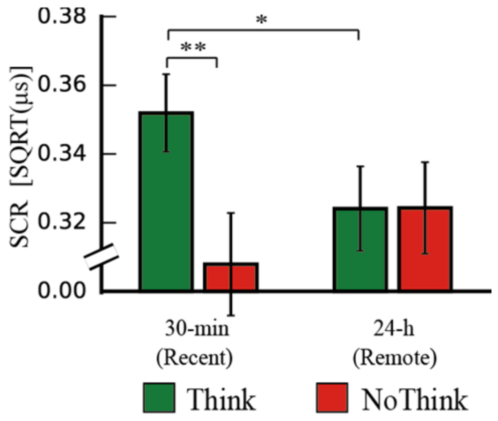

Supplementary Figure 1

Skin conductance response for equalized number of trials. We observed a suppression-induced reduction in SCR for newly acquired memories (T_30min > NT_30min), but not for overnight memories when restricted our analysis only on trials later correctly remembered (T_24h > NT_24h). For "Think" trials, we observed a reduction in SCR for overnight consolidation condition as relative to newly acquired condition. Error bars represent standard error of mean (s.e.m.). Notes: * $p < 0.05$; ** $p < 0.01$.

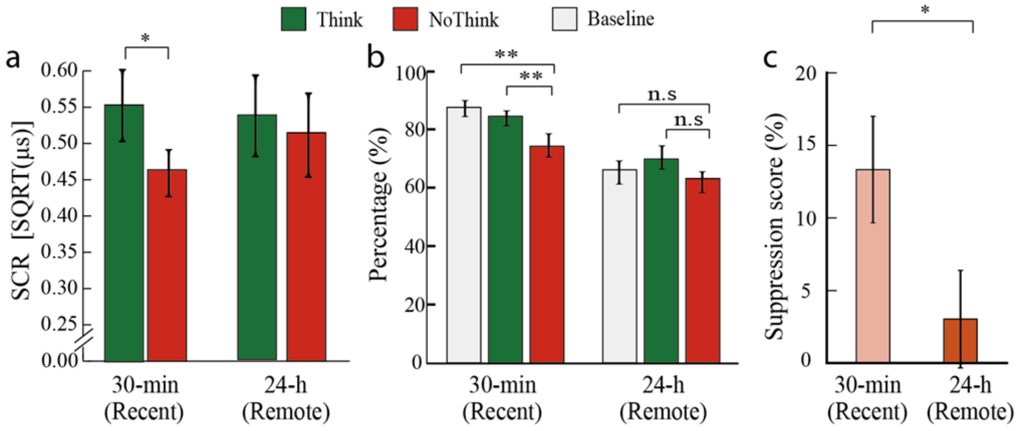

Supplementary Figure 2

Physiological and behavioral results for independent reproducible sample (a) Bar graphs depict SCR associated with retrieval (green) and suppression (red) of aversive memories acquired either 24-h or 30-min prior to the task. **(b)** Bar graphs depict memory performance (i.e., cued-recall accuracy) for newly acquired and overnight consolidated aversive memories as a function of “Think” (green), “NoThink” (red) and “Baseline” (gray) conditions during the testing phase. **(c)** Bar graphs depict suppression scores for both newly acquired and overnight consolidated aversive memories. Error bars represent standard error of mean (s.e.m.). Notes: *$p < 0.05$; **$p < 0.01$.

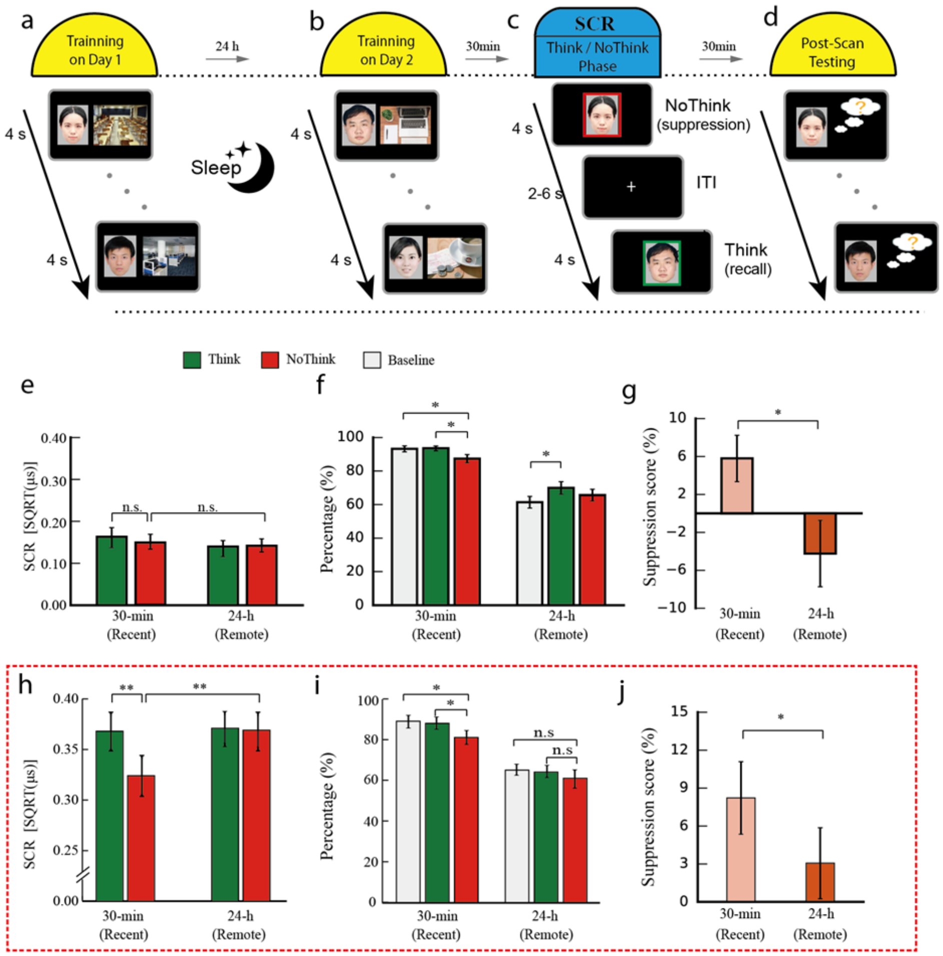

Supplementary Figure 3

Experimental design and results for the behavioral control experiment (**a-b**) The experiment consists of three phases: acquisition, Think/NoThink and post-scan memory test. Same as our original experiment, participants from the neutral control experiment also performed two training sessions on Day 1 and Day 2, which occurred 24-h and 30-min prior to the suppression phase. Participants were trained to memorize 26 pairs in each of the two acquisition sessions (**c**) During the suppression phase; participants were performing a "Think/NoThink" task with concurrent recording of skin conductance responses (SCR). **(d)** During the test phase, participants were given faces as cues and recalled their associated target pictures. **(e)** Bar graphs depict SCR associated with "Think" (green) and "NoThink" (red) conditions for neutral memories acquired either 24-h or 30-min ago. **(f)** Bar graphs depict cued-recall

accuracy for newly acquired and consolidated neutral memories as a function of "Think", "NoThink" and "Baseline" (gray) conditions during the test phase. **(g)** Bar graphs depict suppression scores for newly acquired and overnight neutral memories separately. **(h-j)** Bar graphs depict SCR and cued-recall accuracy for newly acquired and overnight aversive memories in the fMRI study (N = 18). Error bars represent standard error of mean (s.e.m.). Notes: *$p < 0.05$; **$p < 0.01$.

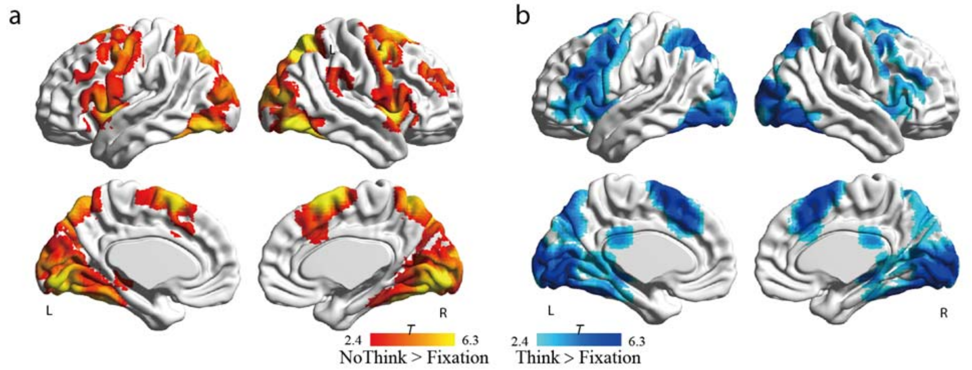

Supplementary Figure 4

Overview of brain systems underlying memory suppression and retrieval (a) Widespread brain activation in prefrontal, parietal and temporal lobes associated with suppression of aversive memories (in red) when compared to the low level passive fixation (also referred to as implicit baseline in the fMRI studies). **(b)** Widespread brain activation in prefrontal, parietal and temporal lobes associated with retrieval of aversive memories (in blue) when compared to passive fixation. Color bars represent *T* values.

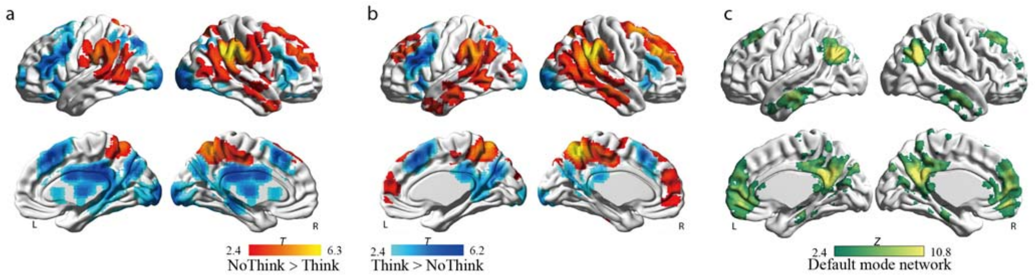

Supplementary Figure 5

Distinct brain systems underlying memory suppression relative to retrieval (a) Widespread brain activation in prefrontal, parietal and temporal lobes associated with suppression (in red, NT_30min > T_30min) and retrieval (in blue, T_30min > NT_30min) of newly acquired aversive memories. **(b)** Widespread brain activation in prefrontal, parietal and temporal lobes associated with suppression (in red, NT_24h > T_24h) and retrieval (in blue, T_24h > NT_24h) of aversive memories after overnight consolidation. Color bars represent *T* values. **(c)** Brain regions constitute of the typical default mode network (DMN) deprived from the most recent meta-analysis of over 11,406 fMRI studies in Neurosynth. Color bar represents *Z* value.

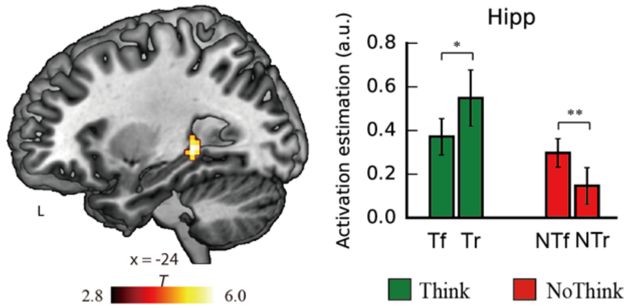

Supplementary Figure 6

Distinct hippocampal activities during intentional and incidental forgetting Sagittal view of significant cluster in the left hippocampus showing interaction effect between suppression and memory status (i.e., NTf - NTr > Tf - Tr). Bar graphs were plotted against the fixation period. Error bars represent standard error of mean (s.e.m.). Notes: * $p < 0.05$; ** $p < 0.01$; Hipp, hippocampus; NTf, NoThink trials that were later forgotten; NTr, NoThink trials that were later remembered; Tf, Think trials that were later forgotten; Tr, Think trials that were later remembered; L, left.

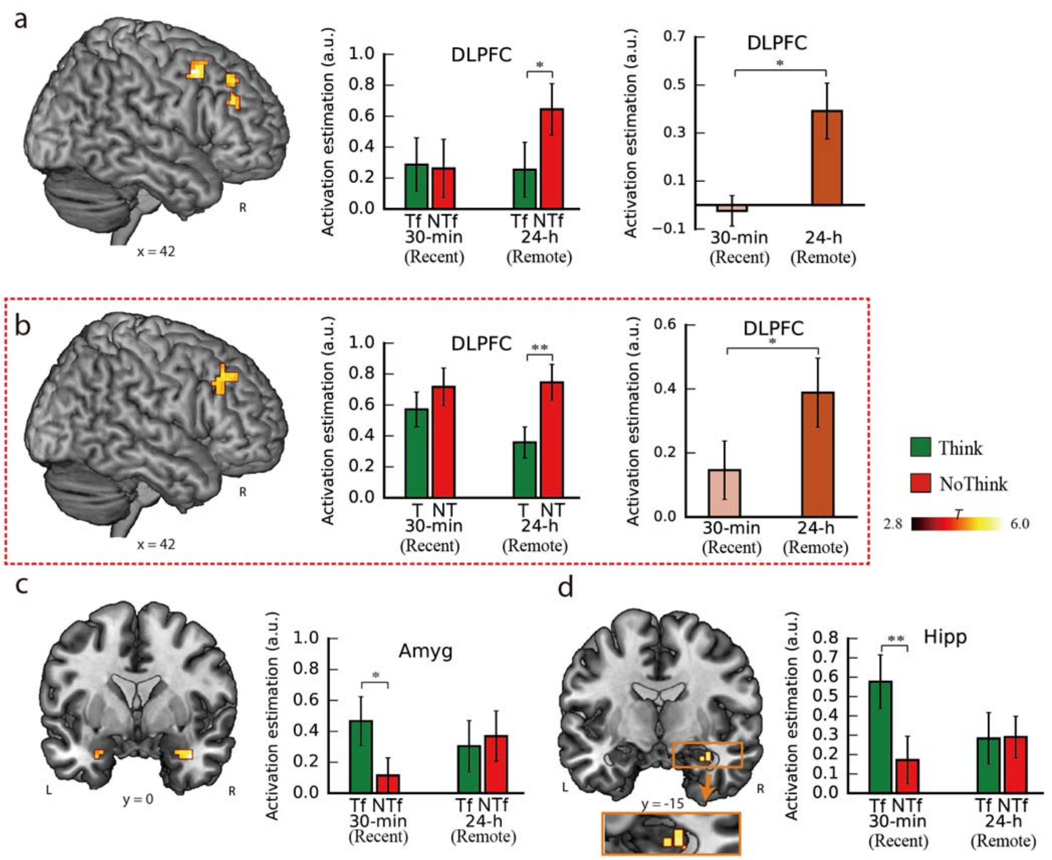

Supplementary Figure 7

Differential prefrontal and hippocampal engagement in intentional forgetting (a) Sagittal view of significant cluster in the right DLPFC showing interaction effect between Time and Suppression (i.e., NTf_24h - Tf_24h > NTf_30min - Tf_30min) for "Think" and "NoThink" trials that were later forgotten. (**b**) Sagittal view of significant cluster in the right DLPFC showing interaction between Time and Suppression in our original results which did not take memory status into account. (**c-d**) Coronal view of significant clusters in the amygdala and hippocampus showing interaction effect between Time and Suppression for "Think" and "NoThink" trials that were later forgotten (i.e., NTf_24h - Tf_24h > NTf_30min - Tf_30min). Bar graphs for "Think" (green) and "NoThink" (red) trials were plotted against fixation period in the Think/NoThink task. Bar graphs in light and dark yellow further indicated the difference between intentional and incidental forgetting separately for newly acquired

(i.e., NTf_30min - Tf_30min) and overnight memories (i.e., NTf_24h - Tf_24h). Color bar represents T values and error bars represent standard error of mean (s.e.m.). Notes: *p < 0.05; **p < 0.01; Hipp, hippocampus; Amy, amygdala; DLPFC, dorsolateral prefrontal cortex; L, left; R, right.

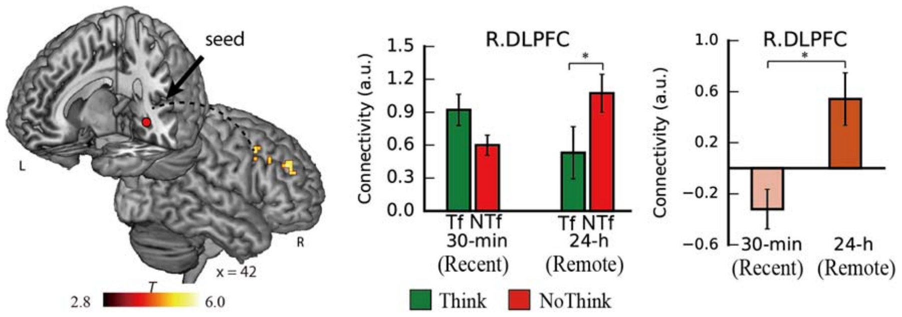

Supplementary Figure 8

Distinct hippocampal-DLPFC functional connectivity in intentional forgetting Hippocampal-DLPFC functional coupling showing significant interaction effect between Time and Suppression for “Think” and “NoThink” trials later forgotten (i.e., NTf_24h - Tf_24h > NTf_30min - Tf_30min). Bar graphs for "Think" (green) and "NoThink" (red) trials were all plotted against the fixation period in the Think/NoThink task. Bar graphs in light and dark yellow further indicated the difference between intentional and incidental forgetting separately for newly acquired (i.e., NTf_30min - Tf_30min) and overnight memories (i.e., NTf_24h - Tf_24h) Color bar represents T values and error bars represent standard error of mean (s.e.m.). Notes: *p < 0.05; Hipp, hippocampus; DLPFC, dorsolateral prefrontal cortex; L, left; R, right.

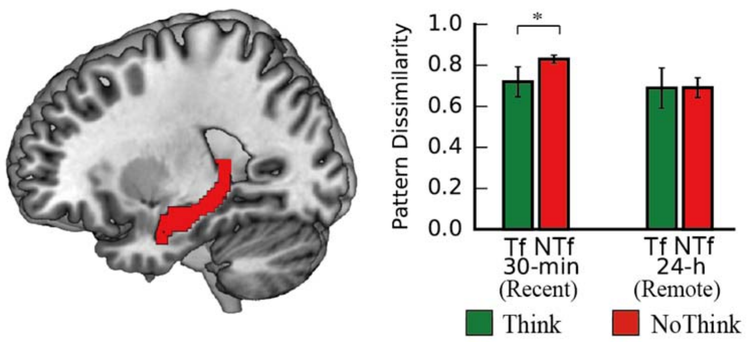

Supplementary Figure 9

Hippocampal pattern dissimilarity for intentional forgetting after consolidation

Higher pattern dissimilarity in the bilateral hippocampus for intentional forgetting (NTf) versus incidental forgetting (Tf) of newly acquired aversive memories, but not for that of overnight aversive memories. Error bars represent standard error of mean (s.e.m.). Notes: *p < 0.05.

Supplemental Tables 1 - 6

Supplementary Table 1

Brain regions involved in recall of aversive memories

Brain Regions	L/R	BA	*T* values	MNI Coordinates (x, y, z)		
			Overnight Consolidated vs. Newly Acquired			
Superior temporal gyrus	R	41	3.07	44	-3	-11
	L		3.21	-59	-29	24
Inferior parietal gyrus	R	33	2.98	59	-37	50
	L		3.01	-58	-35	40
Superior parietal gyrus	R	5	2.88	16	-48	62
	L	2	3.23	-24	-45	66
Angular gyrus	R	5	3.18	9	-46	55
	L		3.06	-3	-37	62
Posterior cingulate gyrus	R	18	3.04	7	-16	43
	L		3.31	-8	-11	42
Middle temporal gyrus	R	41	3.07	42	3	6
			Newly Acquired vs. Overnight Consolidated			
**Hippocampus**	**R**	**-**	**3.52**	**33**	**-39**	**-6**
	**L**	**-**	**3.27**	**-33**	**-33**	**-6**
Caudate	R	-	3.13	13	4	17

Only clusters, significant at a height threshold of $p < 0.01$ and an extent threshold of $p < 0.01$ with family-wise error corrections for multiple comparisons, are reported with local maxima in Montreal Neurological Institute (MNI) space. "-", not applicable. Clusters in the medial temporal lobe are in bold.

Supplementary Table 2

Brain regions involved in suppression of aversive memories

Brain Regions	L/R	BA	*T* values	MNI Coordinates (x, y, z)		
			Newly Acquired: Suppression vs. Retrieval			
**Dorsolateral prefrontal cortex (DLPFC)**	**R**	**9**	**4.10**	**36**	**39**	**42**
			**3.77**	**33**	**45**	**27**
Superior frontal gyrus (SFG)	R	8	3.98	21	24	60
			3.72	9	36	57
Precuneus	R	5	4.74	9	-57	54
	L		4.01	-12	-54	60
Inferior parietal cortex	R	33	6.20	58	-46	40
	L		4.31	-63	-42	42
Superior temporal gyrus	R	35	5.47	57	-44	23
	L	41	4.48	-62	-29	24
Insula	R	41	3.23	45	3	6
			Overnight Consolidated: Suppression vs. Retrieval			
**Dorsolateral prefrontal cortex (DLPFC)**	**R**	**9**	**5.97**	**36**	**39**	**42**
			**4.58**	**27**	**45**	**30**
	**L**		**4.28**	**-33**	**36**	**42**
Superior frontal gyrus (SFG)	R	8	5.17	21	12	54
	L	9	4.21	-27	48	39
		8	3.54	-12	33	54
Precuneus	R	5	6.25	12	-54	51
	L		4.93	-12	-60	60
Inferior parietal cortex	R	33	3.87	56	-46	44
	L		3.32	-54	-46	37
Superior temporal gyrus	R	35	6.17	63	-36	12
	L		5.48	-60	-36	20
Middle temporal gyrus	R	16	5.34	63	-18	11
	L		3.73	-60	-18	-15

Notes are the same as in Supplemental Table 1. Clusters in the dorsolateral prefrontal cortex are in bold.

Supplementary Table 3

Brain regions underlying memory suppression & consolidation interaction

Brain Regions	L/R	BA	*F* values	MNI Coordinates (x, y, z)		
Omnibus *F* contrast: (Suppression vs. Recall) vs. (Newly Acquired vs. Overnight Consolidated)						
**Hippocampus**	**R**	**-**	**15.20**	**36**	**-33**	**-6**
		**-**	**13.17**	**30**	**-33**	**0**
	**L**	**-**	**19.99**	**-33**	**-33**	**-6**
**Amygdala**	**R**	**-**	**9.72**	**21**	**0**	**-12**
	**L**	**-**	**9.88**	**-24**	**0**	**-12**
Caudate	R	-	20.60	18	27	6
		-	14.76	6	21	0
	L	-	14.21	-3	0	21
Putamen	R	-	16.64	30	3	-6
	L	-	14.58	-27	3	-9
Thalamus	R	-	12.95	6	-9	21
	L	-	13.30	0	-12	6
Cerebellum	L	-	10.99	-45	-63	-45
		-	10.41	-36	-63	-39
Dorsolateral prefrontal cortex (DLPFC)	R	9	9.27	27	31	42
Ventral medial prefrontal cortex (VMPFC)	L	10	8.97	-2	56	-4
Inferior temporal cortex	L	15	11.25	-45	-30	-18

Notes are the same as in Supplemental Table 1. Clusters in the medial temporal lobe are in bold.

Supplementary Table 4

Hippocampal functional connectivity changes underlying interaction effects

Brain Regions	L/R	BA	*F* values	MNI Coordinates (x, y, z)		
Omnibus F contrast: (Suppression vs. Recall) vs. (Newly Acquired vs. Overnight Consolidated)						
**Dorsolateral prefrontal cortex (DLPFC)**	**L**	**9**	**11.55**	**-30**	**34**	**41**
			**11.34**	**-34**	**38**	**35**
**Superior frontal gyrus**	**L**	**10**	**13.24**	**-21**	**60**	**6**
			**13.05**	**-21**	**63**	**18**
**Inferior frontal gyrus**	**L**	**41**	**14.56**	**-39**	**24**	**9**
		**38**	**10.82**	**-42**	**45**	**-3**
**Anterior cingulate cortex**	**L**	**26**	**8.78**	**-3**	**42**	**22**
Middle temporal gyrus	R	16	9.93	63	-27	-6
Superior temporal gyrus	L	41	11.65	-51	-30	18
			7.43	-60	-33	15
Inferior parietal gyrus	L	14	13.76	-31	-81	45
Precuneus	L	7	12.00	-9	-66	39
Thalamus	R	-	15.14	9	-30	9
	L	-	9.24	-6	-33	6

Notes are the same as in Supplemental Table 1. Clusters in the prefrontal cortex are in bold.

Supplementary Table 5

Brain regions showing significant representational dissimilarity changes

Brain Regions	L/R	BA	*T* values	MNI Coordinates (x, y, z)		
			Newly Acquired > Overnight Consolidated			
**Hippocampus**	**R**	**-**	**5.98**	**33**	**-36**	**0**
	**L**	**-**	**4.83**	**-33**	**-27**	**-12**
Thalamus	R	-	4.33	18	-18	12
			3.54	21	-14	0
Caudate	R	-	3.35	14	-3	22
			Newly Acquired < Overnight Consolidated			
			None			

Notes are the same as in Supplemental Table 1. Clusters in the medial temporal lobe are in bold.

Supplementary Table 6

Descriptive values of resulting number of trials for the fMRI and SCR analyses

	SCR				fMRI			
	24-h		30-min		24-h		30-min	
	T	NT	T	NT	T	NT	T	NT
Trials (standard)	27 ± 4	24 ± 4	29 ± 3	26 ± 5	36 ± 0	36 ± 0	36 ± 0	36 ± 0
Trials (correctly recalled)	19 ± 5	19 ± 5	19 ± 5	19 ± 5	19 ± 5	19 ± 5	19 ± 5	19 ± 5

The mean ± standard deviation provided in Supplementary Table 6 refers to means across participants.

Supplementary Methods

SCR analysis We first segmented the electrodermal data into event-related time windows based on face onset and then locate face-related SCR amplitude by identifying rises in the electrodermal data which constitute the onset of an SCR. The rises are identified within each time window when at least 5 numbers of continuous points have slope above a predifined threshold (default of 0.0002 μS per second). The trough and peak of candidate SCRs are identified by locating zero crossings in the first-order temporal derivative of the downsampled data. To exclude particularly noisy segments of data (in which an implausible number of candidate SCRs are present), events can be excluded from analysis if more than a specified number of responses are found (default of 8 responses).

To determine if candidate SCRs are likely to contain multiple overlapping responses, inflection points are identified by counting zero crossings in the second-order temporal derivative. If the slope cycles from increase to decrease twice within one candidate SCR, then the overlapping responses are segmented at the inflection point where the slope changes from decrease to increase (in the middle of the rise). Next, candidate SCRs are filtered on the basis of response criteria including: the latency of the SCR relative to the eliciting stimulus (range of 0.5 to 4 seconds); the maximum time between the onset of the SCR and its peak (default of 4 seconds); and the amplitude of the SCR must be above a minimum threshold (default of 0.02 μS). Once plausible SCRs are identified, the peak is determined as the maximum value of the raw data within a buffer centered at the end of the SCR. If multiple SCRs fall within the same window, the largest response is scored.

Multivoxel pattern dissimilarity analysis on forgotten trials only We also performed an additional analysis for "intentional forgetting" (NTf) versus "incidental forgetting" (Tf) items on newly acquired and overnight memories to explore whether suppression would alter multivoxel pattern dissimilarity of associated aversive memories in the hippocampus. This analysis requires at least 2 items within each condition to compute inter-item pattern dissimilarity scores. Six participants were excluded for this analysis due to a lack of at least 2 items in each condition. Paired-*t* tests revealed that intentional forgetting (NTf) is associated with higher hippocampal pattern dissimilarity as compared to incidental forgetting (Tf) for newly acquired memories ($t(11) = 2.21$, $p = 0.031$, $d_{av} = 0.41$), but not for overnight memories ($t(11) = 0.27$, $p = 0.89$, *power* = 0.05) (Supplementary Figure 9). This preliminary result provides some evidence to support the idea that suppression-induced forgetting can indeed alter hippocampal representations of newly acquired memories, but not for overnight consolidated memories.
